# The age-dependent regulation of pancreatic islet landscape is fueled by a HNF1a-immune signaling loop

**DOI:** 10.1016/j.mad.2024.111951

**Published:** 2024-05-31

**Authors:** Andreas Frøslev Mathisen, Thomas Aga Legøy, Ulrik Larsen, Lucas Unger, Shadab Abadpour, Joao A. Paulo, Hanne Scholz, Luiza Ghila, Simona Chera

**Affiliations:** a Mohn Research Center for Diabetes Precision Medicine, Department of Clinical Science, University of Bergen, Bergen, Norway; b Hybrid Technology Hub-Centre of Excellence, Faculty of Medicine, University of Oslo, Norway; c Institute for Surgical Research, Department of Transplant Medicine, Oslo University Hospital, Oslo, Norway; d Department of Cell Biology, Harvard Medical School, Boston, MA, USA

**Keywords:** Pancreatic islets, HNF1A, Pathway analysis, Immune response, Islet ageing

## Abstract

Animal longevity is a function of global vital organ functionality and, consequently, a complex polygenic trait. Yet, monogenic regulators controlling overall or organ-specific ageing exist, owing their conservation to their function in growth and development. Here, by using pathway analysis combined with wet-biology methods on several dynamic timelines, we identified Hnf1a as a novel master regulator of the maturation and ageing in the adult pancreatic islet during the first year of life. Conditional transgenic mice bearing suboptimal levels of this transcription factor in the pancreatic islets displayed age-dependent changes, with a profile echoing precocious maturation. Additionally, the comparative pathway analysis revealed a link between *Hnf1a* age-dependent regulation and immune signaling, which was confirmed in the ageing timeline of an overly immunodeficient mouse model. Last, the global proteome analysis of human islets spanning three decades of life largely backed the age-specific regulation observed in mice. Collectively, our results suggest a novel role of Hnf1a as a monogenic regulator of the maturation and ageing process in the pancreatic islet via a direct or indirect regulatory loop with immune signaling.

## 1. Introduction

Adult development progression and subsequent ageing are multimodal complex processes, involving a wide range of changes impacting general homeostasis, induced by internal or environmental cues (Lopez-Otin et al., 2013). Its cellular and molecular hallmarks are diverse and concurrent, and thus mapping their exact causal network is challenging. The situation is further complicated by the inherent differences in the ageing progression and lifespan between the species. Decisive advances in understanding the molecular biology underlying the age-related processes stem from pioneering studies in classical invertebrate model systems such as *C. elegans*, *Drosophila*, or in the apparently immortal *Hydra* (Brunet, 2020; Tomczyk, 2020). Despite a rather unexpected level of conservation of core ageing mechanisms (Hildegard et al., 2018), the very specific biology and life cycle of these organisms impacted the translatability potential of these findings to mammalian systems, like for example *Sirtuin2* gene (Brenner, 2022). To add to the pain, an increasing body of recent research indicate that, alongside a common molecular backbone, the type and dynamic of the features characterizing adult development and ageing varies between tissues, probably determined by the supplied function (Kaletsky, 2018; Vougioukalaki, 2022). Consequently, mapping the exact chronology of age-related hallmarks requires the independent dynamic molecular mapping of each tissue or organ.

The pancreatic islet is an endocrine organ critical for regulating blood glucose levels. In adult mice, it consists of four main endocrine cell types, each secreting a certain hormone: alpha-cells (glucagon), beta-cells (insulin), delta-cells (somatostatin) and gamma cells (or PP-cells, Ppy). Ageing impacts both the number and functionality of the islet cells (Tuduri, 2022; Chandel et al., 2016). Most of the research focused on the aging of the insulin-secreting beta-cells, with a very limited number of studies addressing the non-beta-cell populations. Although there is a consensus regarding the significant expansion of the human beta-cell mass from birth to adulthood, the effects of ageing are less clear. A study analyzing subjects between 20 and 102 years of age found no significant beta-cell mass changes (Saisho, 2013), while others reported a slight age-related decrease (Rahier et al., 2008; Mizukami, 2014; Talchai et al., 2012; Montanya et al., 2000). In stark contrast, in mice and rats most studies report a significant age-related beta-cell mass increase (Rankin and Kushner, 2009), mostly by increased beta-cell size. Moreover, a similar age-related increase was observed also for the alpha-cell mass (Talchai et al., 2012).

As expected, beta-cell proliferation is age-dependent in both humans and rodents. In humans, it is characterized by a peak during fetal life followed by a steep decline after the second year of life (Gregg, 2012) and a subsequent decrease in the third decade (Arda, 2016). The age-dependent drop in the proliferation rate seems to be conserved in vertebrates being observed in rodents (Rankin and Kushner, 2009; Stolovich-Rain et al., 2012; Lam, 2019; Chen, 2011) or fish (Janjuha, 2018) amongst others, with studies proposing an inverse correlation between beta-cell functionality and proliferative capacity (Klochendler, 2016). Interestingly, experiments in zebrafish showed that the ageing islets display signs of inflammation, which ultimately impede on beta-cell proliferation (Janjuha, 2018).

Gene signatures of inflammageing were also observed in aged rat islets, where an increase in inflammation was reported (Sandovici, 2016). Moreover, the islet T cell population was shown to increase with age in a body-weight independent manner in both mouse and human islets (Denroche et al., 2021). However, there was no significant correlation between immune infiltration (insulitis) and age in the autoimmune form of diabetes (type 1 - T1D) (La Noce et al., 2022). Conversely, the type of insulitic profile influences the age of onset.

At molecular level, age-related changes in human beta- and alpha-cell identity maintenance doubled by transcriptional noise and decreases in certain key beta-cell markers were reported (Mizukami, 2014; Enge, 2017). In contrast, these markers were observed upregulated in mice during the first year of life (Avrahami, 2015).

As the insulin-producing beta-cells are the main producer of insulin in the body, their loss (Thorel, 2010; Chera, 2014) or dysfunction (White et al., 2016) leads to persistent hyperglycemia and diabetes (Eizirik et al., 2020). One of the most prevalent forms of diabetes (type 2 – T2D) is a well-known age-associated disorder (Gunasekaran and Gannon, 2011) (Perl, 2010). Moreover, there is an overlap between organ dysfunction in T2D progression and normal chronological ageing (Palmer et al., 2019) as well as reported insulitis (Boni-Schnetzler et al., 2008). This, combined with the accelerated onset of age-related diseases in diabetes patients, indicates that diabetes can be both a consequence and driver of ageing (Palmer et al., 2019; Xu, 2010; Aronson and Edelman, 2014). Thus, identifying the chronological molecular landscape regulation characterizing the early age-related changes in the pancreatic islet is a must for both calibrating the interventions and interpreting the experimental readouts.

Besides its role in prompting chronic conditions, chronological ageing impacts more subtle processes, such as cell plasticity or regeneration potential. The pancreatic islet is no exception, as chronological changes in the maturation profile, even in young individuals, will trigger highly differential responses. One relevant example is that, even during the first year of life, the murine islet deploys two distinct regenerative mechanisms following total beta-cell loss, according to age. These age-dependent regeneration programs are naturally age-locked and characterized by very different efficiencies, cell types and conversion mechanisms (Thorel, 2010; Chera, 2014). However, the exact dynamic changes in the cellular functions and pathways occurring in the pancreatic islet during the normal maturation process are still largely unknown. Moreover, we know even less about the global regulators controlling these age-related modulations. Consequently, identifying master regulators involved in normal islet ageing will also contribute to unlocking the mechanisms governing plasticity potential.

Yet, with few exceptions, most high-throughput studies focused on the important issue of murine islet ageing, thus employing comparisons with senescent animals (Almaca, 2014; Aguayo-Mazzucato, 2017; Arrojo, 2019), and therefore having reduced resolution on the overall dynamic of the progression of adult postnatal development fingerprint. Thus, a high-resolution mapping of the cellular functions and pathways driving the pancreatic islet during chronological postnatal development and ageing is still largely missing. Moreover, even less is known about the global regulators controlling these age-related modulations.

To bridge this gap, here we constructed several multipoint chronological timelines of murine islet first year postnatal development. By combining transgenic systems, global transcriptomics, pathway analysis, cell biology and physiology, we mapped the age-specific cellular and molecular changes characterizing the gradual transition from young to ageing mice as well as identified *Hnf1a* (*Hepatocyte nuclear factor 1-alpha*) as a novel master regulator of pancreatic islet maturation modulating a critical subset of these processes.

*Hnf1a* is a homeobox transcriptional activator crucial for both liver and pancreatic development (Boj et al., 2001; Odom, 2004; Servitja, 2009). Mutations in *Hnf1a* are responsible for HNF1A-MODY (MODY3 – Maturity Onset Diabetes of the Young), the most prevalent form of monogenic diabetes. The disorder presents an autosomal dominant inheritance pattern (e.g. heterozygous individuals are affected) and, interestingly, displays an age-related penetrance, the patients usually developing the disease in early adulthood. Moreover, a common HNF1A variant was previously associated with significantly decreased diagnosis age (Locke, 2018).

Of note, certain HNF1A mutations, that do not cause HNF1A-MODY, significantly increase T2D risk (Wiltshire, 2004; Najmi, 2017) in an age specific manner, being usually characterized by an earlier age of onset (Li et al., 2022). Three HNF1A variants were particularly related to increased risk for T2D, including two non-synonymous variants and one inter-genic single nucleotide polymorphism (SNP) (Gaulton, 2015).

## 2. Materials and methods

### 2.1. Murine models

Animal work was approved and performed according to the regulations of the Norwegian Animal Research Authority (authorization numbers FOTS 8423, 10785, 12105, 20547, 25526, 25531). All mice were fed ad libitum with standard diet RM1A (SDS) with free access to water and kept in individual ventilated cages at 22°C in a 12-hour light/dark cycle.

The following mouse strains were used C57BL6/J mice (mixed background) and NOD.*Cg-Prkdc*^*scid*^*Il2rg*^*tm1Wjl*^ Tg (Ins2-HBEGF) 6832Ugfm/Sz (termed NSG) (Yang, 2015; Furuyama, 2019). *Hnf1a*^*flox/flox*^ (termed Hnf1a^HMZ^) mice were generated by Ozgene Pty Ltd (Bentley WA, Australia). The targeting construct was electroporated into a C57BL/6 ES cell line, Bruce4 (Kontgen et al., 1993). Homologous recombinant ES cell clones were identified by Southern hybridization and injected into goGermline blastocysts (Koentgen, 2016). Male chimeric mice were obtained and crossed to ubiquitous Flp deleter females to remove the neomycin selection cassette. The resulting knock-in line introduced loxP sites flanking exon 4 of the murine *Hnf1a* gene. Upon Cre-recombination, exon4 is deleted, removing the IPR001356 homeobox domain, and producing a non-functional downstream sequence. Experimental mouse strain Hnf1a^HTZ^ was generated by crossing the *Hnf1a*^*HMZ*^ strain with *RIP-Cre* mice (Herrera, 2000), generously provided by Prof. Pedro Herrera. Experimental mice were identified by genotyping using the following primers for the *Hnf1a* flox allele: 5’-AACCACCCTCTCTCCCAGTAAG-3’(forward) and 5’-GTGTGTGTAACCGGAGTAGAAG-3’(reverse).

### 2.2. Assessment of blood glucose, intra-peritoneal glucose tolerance test and insulin levels

Blood glucose was measured bi-weekly using a Contour XT glucometer (Bayer) at set time-points. For the glucose tolerance testing, mice were fasted for 15 hours over night, before receiving an intraperitoneal injection at 2.0 g/kg of D-glucose (Sigma). Glycaemia was measured at 5, 10, 15, 20, 30, 45, 60, 75, 90, 115, and 120 min following the injection. Blood from the mice were collected in Lithium Heparin Microvette CB 300 tubes with subsequent serum preparation by centrifugation at 4°C, 14000 g for 10 minutes. Serum insulin levels were measured using the Mercodia Ultrasensitive Insulin ELISA kit (10–1132–01, Mercodia).

### 2.3. Immunofluorescence staining and imaging

Mouse pancreases were collected and processed as described previously (Legøy, 2020) with a fixation in 4 % PFA for 2 hours at RT, followed by successive incubations of 10, 20, 30 % sucrose, then embedded in Tissue Tek OCT compound (Sakura JP). Cryosections of 10 μM thickness were sliced using a cryotome (Leica CM 1950, Leica) and placed on SuperFrost Plus slides (Thermo Scientific). The following primary antibodies were used: mouse anti-porcine glucagon (1/1000, G2654, Sigma-Aldrich), guinea-pig anti-porcine insulin (1/400, A056401–2, Dako), rat-anti somatostatin (1:100, sc-47706 Santa Cruz), guinea pig anti-glucagon (1:400, AK247, Geneva antibody facility), mouse IgG1 anti-pancreatic polypeptide (1:300, MAB62971, R&D systems), rabbit anti-somatostatin (1:500, ab111912, Abcam), and rabbit anti-Hnf1a (1:100 ab204306, Abcam). The following secondary antibodies were used: goat anti-guinea pig (A546, A488), goat anti-mouse IgG1 (A647, A546), goat anti-Rat IgG (A546), goat anti-rabbit (A647) all used at a concentration of 1:500 and from Molecular Probes. DAPI (1:1000, D1306, Molecular probes) was used for nuclear stain. Prolong diamond antifade Mounting media (P36970, Life technologies) was used to mount the stained slides. Heat retrieval was used for the nuclear staining of HNF1A, by heating for 5 min in pH 6.0 10 mM citrate buffer using a pressure cooker (Ninja).

Image acquisition was performed on either Andor Dragonfly 505 confocal microscope (Oxford instruments), Leica TCS SP5 STED CW or Leica TCS SP8 STED 3X (Leica Microsystems, CMS gmbh). Some minor artefacts are known to randomly occur in the panoramic images stitching generated by the Andor Dragonfly confocal, thus volume segmentation and cell counting was never performed cross-instruments for the comparison of the same parameter in different conditions.

### 2.4. Volume segmentation and cell counting

Images from the Andor dragonfly were analysed using the accompanying Imaris software (Bitplane), version 9.1.2. For the segmentation of insulin volumes, the imaris surface segmentation algorithm was used at 2,0μm resolution, segmenting based on absolute signal intensity. Each surface was manually fit to the stained insulin area.

Cell counting was performed manually either in Imaris or using LASX (Leica) for images from the SP8 microscope. For supervised automated counting of HNF1A stained nuclei, FIJI (imageJ version 2.9.0) was used. Thresholding was done with “AutoThreshold” using Otsu Dark method on Max projection images from the SP8. Masks were then generated before a ROI was manually drawn around the islet. The number of cells was then counted by the “Analyse particles feature” with a size gating of 10μm.

### 2.5. Pancreatic Islet isolation

Pancreatic islets were isolated by injecting 0.2 % collagenase solution (Sigma Aldrich, C7657) into the common bile duct before the pancreas was collected and digested for 9 minutes on a water bath at 37°C. After subsequent washes, the islets were separated using a Histopaque gradient (Histopaque 1119, Sigma Aldrich 11191) of 1.119, 1.110, 1.100, and 1.080 g/mL. Islets were manually collected and washed before further processing.

### 2.6. RNA sequencing

Total RNA was prepared using RNeasy Mini Kit (Qiagen). The amount and quality of the purified RNA was measured with a NanodropOne (Thermo Scientific), before samples were shipped to Qiagen NGS-Centre for sequencing and analysis. The Qiagen Genomic Facility performed a second quality control, library preparation, sequencing, and mapping. Briefly, adapter and quality trimming was done by “Trim Reads” tool from CLC Genomics Workbench. Further, reads were trimmed based on quality scores and ambiguous nucleotides, e.g. due to stretches of Ns. A maximum of 2 ambiguous nucleotides were allowed in a read. The QC reports were generated by “QC for Sequencing Reads” tool from CLC Genomics Workbench. Read mapping and gene quantification were done by “RNA-seq Analysis” tool from CLC Genomics Workbench. The ‘Empirical analysis of DGE’ algorithm of the CLC Genomics Server 11.0.3 was used by Qiagen bioinformatics department or by us for differential expression analysis with default settings. It is an implementation of the ‘Exact Test’ for two-group comparisons developed by Robinson and Smyth (Robinson and Smyth, 2008) and incorporated in the EdgeR Bioconductor package (Robinson et al., 2010). Fold changes were calculated from the generalized linear model, which corrects for differences in library size between the samples and the effects of confounding factors. To account for multiple testing problem, “FDR p-values” were calculated using the Benjamini-Hochberg method. Normalized expression values (TPM) for each gene and sample were calculated.

The full datasets were deposited to the NCBI Gene Expression Omnibus repository, accession numbers GSE193327, GSE193403 and GSE231710.

### 2.7. Data and pathway analyses

Differentially expressed gene lists were generated using the CLC genomics workbench (Qiagen), see above. The gene lists (FC ≥ 1.5, p < 0.05) were then uploaded to the Ingenuity Pathway Analysis (IPA, Qiagen) software and used for expression core and comparison analyses with following parameters: Interaction networks: 35 molecules per network, 25 networks per analysis, causal Networks active; node types: all entities except chemical subclasses; Data sources: All, Species: mouse / human (according to the dataset origin).

Venn clustering of differentially expressed genes were performed with Venny version 2.1 (BioinfoGP).

### 2.8. Human islets sources and ethical statements

The Norwegian Regional Committee of Medical and Health Research Ethics approved the reported experimental protocols used for human islets (REK 2011/426 to Hanne Scholz). All methods were carried out in accordance with the Helsinki Declaration and informed consent was obtained from the relatives (organ donations). Human islets were isolated as previously described (Friberg et al., 2008), here from twelve donors, men and women (age between 35 and 67 years old) (Table S1). Briefly, human pancreas were obtained from brain-dead non-diabetic donors, and digested by collagenase solution. Islets were collected following a Ficoll gradient separation. In this study, one of the human islet samples was excluded because of technical issues (duplicated impure sample).

### 2.9. Global proteomics in human islets

Human islets were processed as previously described (Mathisen, 2023), being lysed in 4 % SDS buffer on a shaker at 95°C for 7 min, followed by sonication. The protein concentration was determined using a BCA protein assay kit (Thermo Fisher Scientific, Waltham, MA, USA, catalog number 23225). 100 μg of dry proteins were processed by Filter-Aided Sample Preparation method.

#### 2.9.1. Tandem mass Tag (TMTpro) labelling

TMTpro16 reagents were re-suspended in ACN. Desalted peptides were re-suspended in 50 μL of 200 mM HEPES pH 8.5, 15 μL of ACN, and 7 μL of the TMT reagents were added to the respective peptide samples, gently vortexed, and incubated for 1 h at RT. To prevent unwanted labelling, the reaction was quenched by adding 10 μL of 5 % hydroxylamine and incubated for 15 min at RT. Equal amounts of the TMT-labelled samples were combined and concentrated to near dryness, followed by desalting via C18 solid phase extraction.

#### 2.9.2. Off-line basic pH reversed-phase (BPRP) fractionation

We fractionated the pooled, labeled peptide sample using BPRP HPLC (Wang, 2011). We used an Agilent 1200 pump equipped with a degasser and a detector (set at 220 and 280 nm wavelength). Peptides were subjected to a 50-min linear gradient from 5 % to 35 % acetonitrile in 10 mM ammonium bicarbonate pH 8 at a flow rate of 0.6 mL/min over an Agilent 300Extend C18 column (3.5 μm particles, 4.6 mm ID and 220 mm in length). The peptide mixture was fractionated into a total of 96 fractions, which were consolidated into 24 super-fractions (Paulo, 2016). Samples were subsequently acidified with 1 % formic acid and vacuum centrifuged to near dryness. Each consolidated fraction was desalted via StageTip, dried again via vacuum centrifugation, and reconstituted in 5 % acetonitrile, 5 % formic acid for LC-MS/MS processing.

#### 2.9.3. LC-MS/MS-analysis

From each of 12 non-adjacent fractions, ~5 μg was dissolved in 5 % aqueous formic acid (FA) /5 % acetonitrile prior to LC-MS/MS analysis on an Orbitrap Eclipse mass spectrometer (Thermo Fisher Scientific, San Jose, CA) coupled to a Neo Vanquish liquid chromatography (LC) pump (Thermo Fisher Scientific). Peptides were fractionated on a 100-μm inner diameter microcapillary column packed with ~35 cm of Accucore resin (2.6 μm, 150 Å, ThermoFisher Scientific). For each analysis, we loaded ~1 μg onto the column.

The scan sequence began with an MS1 spectrum (Orbitrap analysis, resolution 60,000, 350–1350 Th, automatic gain control (AGC) target 4×105, maximum injection time 50 ms). The hrMS2 stage consisted of fragmentation by higher energy collisional dissociation (HCD, normalized collision energy 36 %) and analysis using the Orbitrap (AGC 200 %, maximum injection time 86 ms, isolation window 0.6 Th, resolution 50,000). Data were acquired using the FAIMSpro interface the dispersion voltage (DV) set to 5000 V, the compensation voltages (CVs) were set at −40 V, −60 V, and −80 V, and the TopSpeed parameter was set at 1 sec per CV.

#### 2.9.4. MS data analysis

Mass spectra were processed using a Comet-based in-house software pipeline (Huttlin, 2010), and spectra were converted to mzXML using a modified version of ReAdW.exe. Database searching included all entries from the human Uniprot database (March, 2021). This database was concatenated with one composed of all protein sequences in the reversed order. Searches were performed using a 50 ppm precursor ion tolerance for total protein level analysis. The product ion tolerance was set to 0.9 Da. These wide mass tolerance windows were chosen to maximize sensitivity in conjunction with Sequest searches and linear discriminant analysis (Huttlin, 2010; Beausoleil et al., 2006). TMTpro tags on lysine residues and peptide N termini (+304.207) and carbamidomethylation of cysteine residues (+57.021 Da) were set as static modifications, while oxidation of methionine residues (+15.995 Da) was set as a variable modification.

Peptide-spectrum matches (PSMs) were adjusted to a 1 % false discovery rate (FDR) (Elias and Gygi, 2007; Elias and Gygi, 2010). PSM filtering was performed using a linear discriminant analysis, as described previously (Huttlin, 2010), while considering the following parameters: XCorr, ΔCn, missed cleavages, peptide length, charge state, and precursor mass accuracy. For TMT-based reporter ion quantitation, we extracted the summed signal-to-noise (S/N) ratio for each TMT channel and found the closest matching centroid to the expected mass of the TMT reporter ion.

The search space for each reporter ion was limited to a range of 0.003 *m/z* to prevent overlap between the isobaric reporter ions. For protein-level comparisons, PSMs were identified, quantified, and collapsed to a 1 % peptide false discovery rate (FDR) and then collapsed further to a final protein-level FDR of 1 %. Moreover, protein assembly was guided by principles of parsimony to produce the smallest set of proteins necessary to account for all observed peptides.

Proteins were quantified by summing reporter ion counts across all matching PSMs using in-house software, as described previously (Huttlin, 2010). PSMs with poor quality, MS3 spectra with TMT reporter summed signal-to-noise ratio that is less than 100, or no MS3 spectra were excluded from quantitation (McAlister, 2012). Protein quantitation values were exported for further analysis in Microsoft Excel and GraphPad Prism (version 8). Each reporter ion channel was summed across all quantified proteins and normalized assuming equal protein loading of all samples.

The proteomics data have been deposited to the ProteomeXchange Consortium via the PRIDE partner repository (Perez-Riverol, 2019) with the dataset identifier PXD041619.

### 2.10. Statistical analysis

Statistical analyses were performed using GraphPad Prism v9.5.1 (GraphPad Software Inc., USA). RNAseq data were generated with the “Empirical analysis of DGE’ algorithm in the CLC Genomics Workbench. FDR corrected p-values were used, with a p-value of ≤0.05 being considered the significance threshold. Statistical analysis on the proteomics data was performed using unpaired two-tailed Student’s t test and a p-value of ≤0.05 was considered significant. To assess the statistical differences between groups for the immunofluorescence data quantification and physiological parameters we used non-parametric Mann Whitney test, unless otherwise specified. Area under the curve was used for the glucose tolerance and long-term glycemia curves. In figures, data are represented as mean ± SD (standard deviation) unless otherwise specified. Statistical significance was defined at P < 0.05 (*), P < 0.01 (**), P < 0.001 (***), and P < 0.0001 (****).

## 3. Results

**Fig. 1.**
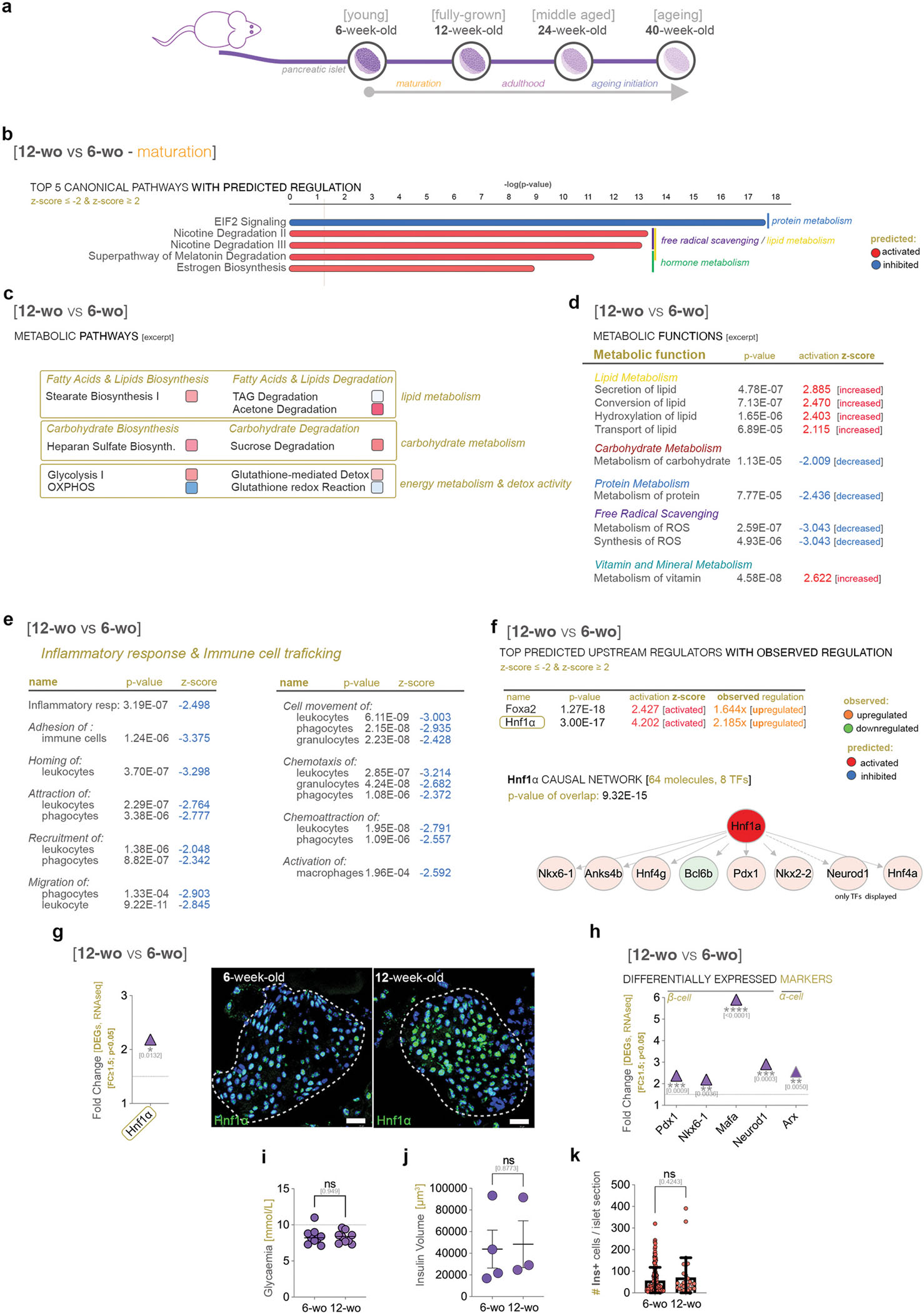
The islet transcriptional landscape characterizing the normal maturation stage based on the differentially regulated landscape between fully-grown and young adults. a) Scheme illustrating the age timeline with the four points of analysis; b) IPA-generated top 5 canonical pathways with predicted activation pattern (z-score ≥ 2 – activated – red; z-score ≤ −2 – inhibited - blue); c) Metabolic pathways with predicted activation pattern (z-score ≥ 2 – activated – red; z-score ≤ −2 – inhibited - blue); d) Metabolic functions with predicted activation pattern (z-score ≥ 2 – activated; z-score ≤ −2 – inhibited); e) Immune response and immune trafficking processes with activity pattern based on the differential transcriptional landscape between fully-grown and young mice; f) Top predicted upstream regulators with observed differential expression and IPA-generated *Hnf1a* causal network (red – activated; orange – upregulated; green – downregulated); g) Graph displaying the observed statistically significant upregulation FC ≥ 1.5, p < 0.05) of *Hnf1a* in the RNAseq dataset and immunofluorescence images of Hnf1*α* staining in young and fully-grown adults (scale – 20 μm, green – Hnf1*α*); h) Graph displaying the observed upregulation (FC ≥ 1.5, p < 0.05) of selected differentially expressed alpha- and beta-cell markers based on RNAseq data; i) Graph depicting the recorded glycemia in 6- and 12-week-old mice (non-parametric Mann-Whitney test, each data point represents one distinct animal); j) Automated insulin volume quantification based on Imaris rendering of insulin signal in 6- and 12-week-old mice (unpaired t-test with Welch’s correction, each data point represents one distinct animal, an average of 26 islets were assessed / mouse; k) Graph depicting the manual quantification of the number of insulin positive (Ins+) cells per islet section in 6- and 12-week-old mice (unpaired t-test with Welch’s correction, each data point represents one distinct islet section, at least 3 mice were analyzed per condition) *p<0.05, **p<0.01, ***p<0.001, ****p<0.0001).

To identify the chronology of the age-related molecular processes in mice with a heterogeneous background, we performed transcriptomic analysis on isolated pancreatic islets at different ages (Fig. 1a). To avoid the strong interference of sexual maturation cues, all time points were collected post puberty and corresponded to: (i) young (*6-week-old* – not fully matured); (ii) fully-grown (*12-week-old*, 8–12 week-old being the standard adult timepoint for most experiments); (iii) middle-aged (*24-week-old -* some detectable signs of physical function decline) and ageing adults (*40-week-old* - possible presence of senescence changes).

### 3.1 The transcriptional landscape during the maturation stage in mice is governed by key islet regulators and reveals a tilt of the energy homeostasis towards increased lipid metabolism

We first focused on mapping the molecular changes characterizing the transition from young to fully-grown adult (maturation stage). To comprehensively assess this, we performed pathway analysis using IPA (Ingenuity Pathway Analysis software, Qiagen) on the differentially expressed genes (DEGs, FC≥1.5, p<0.05) delineating this final growth period. The analysis indicated signaling involved in protein, lipid, carbohydrate, and free radical scavenging metabolism in the top pathways with predicted regulation (Fig. 1b, c). Consistently, Lipid Metabolism and Carbohydrate metabolism were inferred in the top cellular and molecular functions of the analyzed transcriptional landscape (Supp. Fig. 1a).

The detailed functions analysis indicated the decrease of several key metabolic and cellular processes in fully-grown adults, such as the metabolism of carbohydrate, protein, and reactive oxygen species (ROS) amongst others (Fig. 1d). Of note, the same descending trend characterized a large number of immune functions involved in the inflammatory response and cell trafficking (Fig. 1e, Supp. Fig. 1b). In contrast, other metabolic processes, especially the metabolism of lipids and vitamin, were predicted to be increased based on the differential transcriptional landscape (Fig. 1d). Taken together, these results suggest a decrease in the carbohydrate and protein turnover, coupled with an increase in lipid metabolism during this final period of maturation, with fully-grown adults also presenting a lower ROS generation than their younger (6-week-old) counterparts.

To identify the master regulators responsible for these changes, we inspected the top predicted upstream regulators and focused on the ones observed to be also significantly deregulated (FC≥1.5, p<0.05) during this period in the dataset (Fig. 1 f). The top two hits, *Foxa2* and *Hnf1a* represented key genes involved in pancreas development as well as in islet cell identity and function (Cujba, 2022; Haliyur, 2019; Wang et al., 1998; Servitja, 2009). Of note, heterozygous mutations in human HNF1A gene cause HNF1A-MODY (or MODY3), the most prevalent form of monogenic maturity onset diabetes (Locke, 2018; Yamagata, 1996; Bjorkhaug, 2003). Besides being predicted as an upstream regulator of the analyzed transcriptional landscape, *Hnf1a* was indeed observed significantly upregulated (*2.185x, p*=*0.0132*) in fully-grown adults as well as detected in high levels by immunostaining in these islets (Fig. 1 g).

Due to its role in pancreas development, we specifically assessed genes involved in islet cell identity, including key markers of beta-cells (*Pdx1* [*p*=*0.0009*]*, Mafa* [*p*=*0.0036*]*, Nkx6.1* [*p*<*0.0001*], and *Neurod1* [*p*=*0.0003*]) and alpha-cells fate (*Arx, p*=*0.0050*) and found that these followed the same regulation as *Hnf1a*, being significantly upregulated in fully-grown adults (Fig. 1 h). Of note, despite these increased expression levels, the islet hormone regulation (Supp Fig. 1c *Ins2* [*p*=*0.4665*]*, Gcg* [*p*=*0.9752*]*, Sst* [*p*=*0.1155*]*, and Ppy* [*p*=*0.5564*]), glycemia (Fig. 1i, *p*=*0.9490*), total beta-cell volume (Fig. 1j, *p*=*0.8773,* Supp. Fig. 1d) or number of Ins+ cells / islet section (Fig. 1k, *p*=*0.4243*) were unchanged. Overall, these results suggest a role for *Hnf1a* in the regulation of the maturation stage in mice.

### 3.1. The regulatory pattern characterizing fully-grown adults is age-restricted and transient

**Fig. 2.**
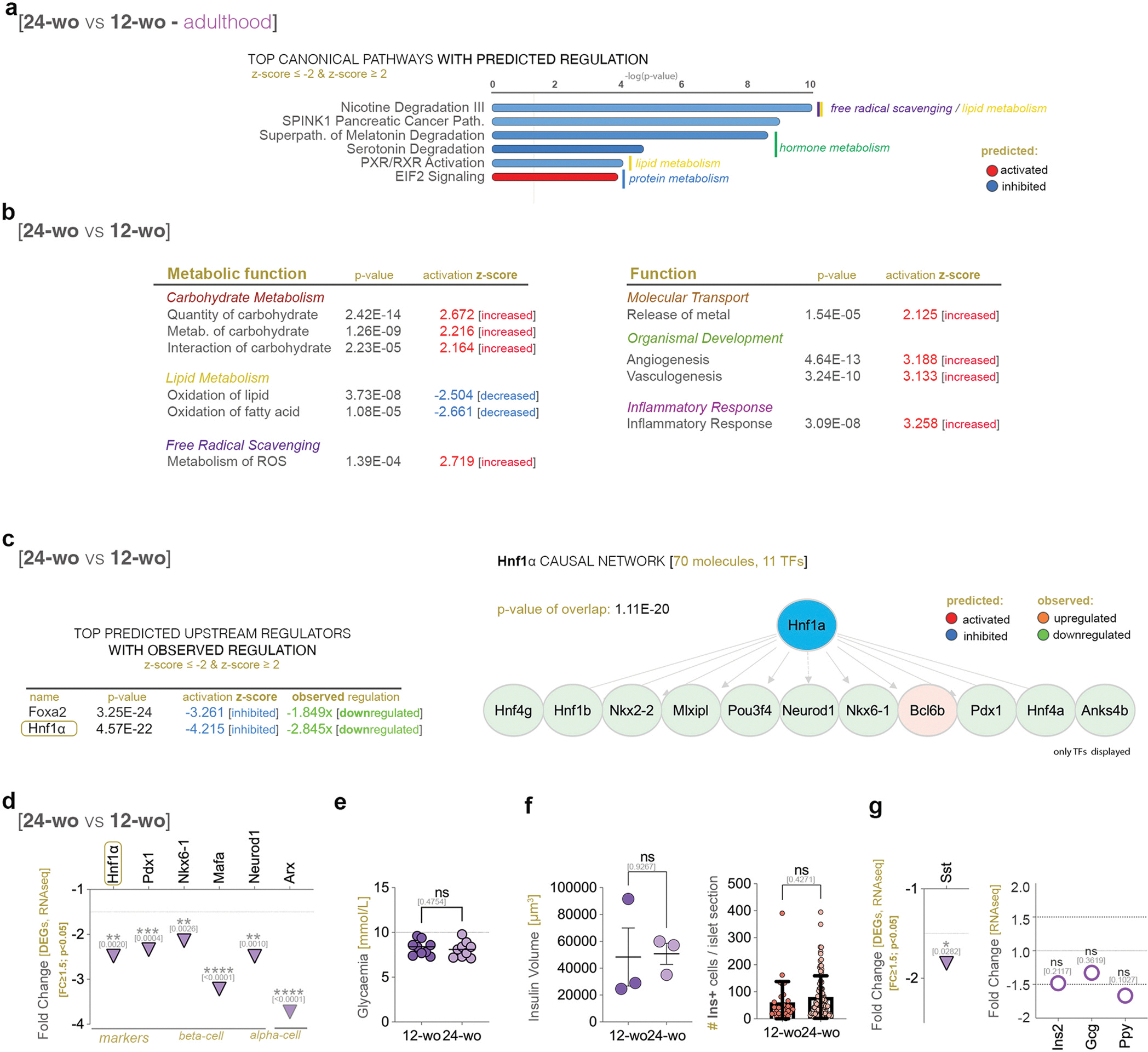
The islet transcriptional landscape during adulthood, based on the differentially regulated landscape between middle-aged and fully-grown adults. a) Top 5 canonical pathways with predicted activation pattern (z-score ≥ 2 – activated – red; z-score ≤ −2 – inhibited - blue); b) Metabolic functions with predicted activation pattern (z-score ≥ 2 – activated; z-score ≤ −2 – inhibited) characterizing the adulthood stage; c) Top predicted upstream regulators with observed differential expression and IPA-generated *Hnf1a* causal network (red – activated; orange – upregulated; green – downregulated); d) Graph displaying the observed downregulation of *Hnf1a* and selected differentially expressed alpha- and beta-cell markers based on RNAseq data (FC ≥ 1.5, p < 0.05); e) Graph depicting the recorded glycemia in 12- and 24-week-old mice (non-parametric Mann-Whitney test, each data point represents one distinct animal) f) Graphs depicting the automated insulin volume quantification (unpaired t-test with Welch’s correction, each data point represents one distinct animal, an average of 26 islets were assessed / mouse) and manual quantification of the number of insulin positive (Ins+) cells per islet section (unpaired t-test with Welch’s correction, each data point represents one distinct islet section, at least 3 mice were analyzed per condition) in 12- and 24-week-old mice; g) Graphs displaying the observed statistically significant (FC ≥ 1.5, p < 0.05) *Sst* downregulation and the unchanged regulation of the other islet hormones in the RNAseq dataset. (*p<0.05, **p<0.01, ***p<0.001, ****p<0.0001).

We further mapped the transcriptional changes occurring during adulthood, i.e., between 12 and 24 weeks. The pathway analysis revealed signaling involved in lipid metabolism and free radical scavenging, however presenting an opposite pattern of activation than during the previous stage (compare Fig. 2a and Fig. 1b), suggesting only a transient regulation of these pathways in fully-grown adults.

Consistent with the change in the regulatory pattern of the top signaling pathways, the analysis indicated a switch in the regulation of several metabolic functions as well as a diversification of deregulated processes as compared to the maturation stage (compare Fig. 2b with Fig. 1d and Supp. Fig. 1b). Amongst these, the metabolism of carbohydrate and ROS, were inferred increased in middle-aged adults. These data suggest changes in the energy metabolism leading to accumulation of reactive oxygen species with the progression of adulthood.

Of note, *Hnf1a*was again inferred in the top predicted upstream regulators, being also observed significantly downregulated *(−2.48x, p*=*0.0020*) in middle-aged adults (Fig. 2c). Similarly, islet cell identity markers were significantly downregulated (Fig. 2d), however without a significant impact on glycemia regulation (Fig. 2e *p*=*0.4754)* or total beta-cell volume (Fig. 2 f *p*=*0.9267*). Of interest, one of the main islet hormones, *Somatostatin* (*Sst*), was also significantly downregulated in middle-aged mice *(−1.834x, p*=*0.0282*), while *Insulin 2* (*Ins2* - the main insulin gene in mice, *p*=*0.2117*), *Glucagon* (*Gcg, p*=*0.3619*) and *Pancreatic polypeptide (Ppy, p*=*0.1027)* transcripts were unchanged (Fig. 2 g).

### 3.2. Hormone secretion, carbohydrate and energy metabolism are impacted during ageing initiation

**Fig. 3.**
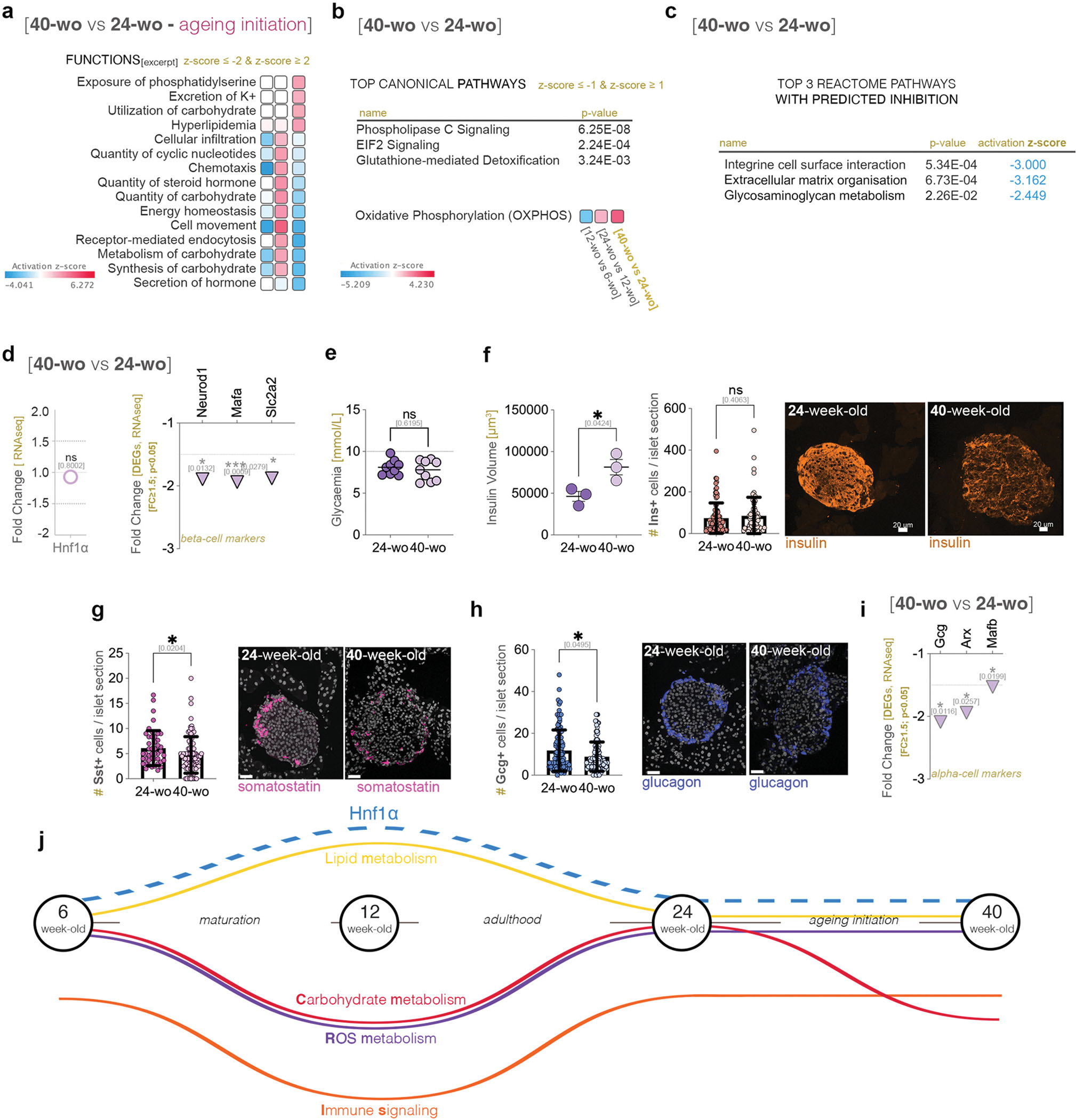
The islet transcriptional landscape characterizing the ageing initiation stage based on the differentially regulated landscape between ageing and middle-aged adults. a) Comparison analysis of the molecular and cellular functions activity pattern between the different stages (z-score ≥ 2 – activated – red; z-score ≤ −2 – inhibited - blue); b) Top canonical pathways of the analyzed transcriptional landscape; c) Top Reactome pathways with inferred inhibition; d) Graphs of the observed regulation of *Hnf1a* and selected beta-cell markers during ageing initiation, based on RNAseq data (FC ≥ 1.5, p < 0.05); e) Graph depicting the recorded glycemia in 24- and 40-week-old mice (unpaired t-test with Welch’s correction, each data point represents one distinct animal); f) Graphs depicting the automated insulin volume quantification (non-parametric Mann-Whitney test, each data point represents one distinct animal, an average of 22 islets were assessed / mouse; scale – 20 μm; orange - insulin) and manual quantification of the number of insulin positive (Ins+) cells per islet section (unpaired t-test with Welch’s correction, each data point represents one distinct islet section, at least 3 mice were analyzed per condition) between middle-aged and ageing adults as well as representative immunofluorescence images (scale – 20 μm); g) Graph depicting the manual quantification of the number of somatostatin positive (Sst+) cells per islet section (non-parametric Mann-Whitney test, each data point represents one distinct islet section, an average of 50 islets were assessed / mouse; and 3 mice were analyzed per condition) and representative immunofluorescence images of Sst staining in middle-aged and ageing adults (scale – 20 μm, light blue – somatostatin); h) Graph depicting the manual quantification of the number of glucagon positive (Gcg+) cells per islet section (non-parametric Mann-Whitney test, each data point represents one distinct islet section, an average of 44 islets were assessed / mouse; and 3 mice were analyzed per condition) and representative immunofluorescence images of Sst staining in middle-aged and ageing adults (scale – 20 μm, blue – glucagon). i) Graph displaying the observed statistically significant downregulation of *Gcg* and key alpha cell markers in the RNAseq dataset (FC ≥ 1.5, p < 0.05); j) Overview scheme of the main regulatory activity patterns and Hnf1a regulation. (*p<0.05, **p<0.01, ***p<0.001, ****p<0.0001).

The comparison pathway analysis between middle-aged and ageing mice indicated, amongst others, a decrease in the carbohydrate metabolism, hormone secretion and energy metabolism, and an increase in the hyperlipidemia and excretion of K+ (Fig. 3a, Supp Fig. 1e), while other functions, such as oxidation of fatty acids or ROS metabolism were unchanged as compared to the previous stage (Supp Fig. 1 f).

The pathway analysis indicated signaling involved in protein, lipid, and energy metabolism in the top canonical pathways (Fig. 3b). Of note, signaling involved in energy production, such as OXPHOS was predicted activated (activation z-score: 2.449) in ageing mice as compared to their middle-aged counterparts. In contrast, in the top reactome pathways with inferred inhibition were signaling involved in extracellular matrix organization and carbohydrate metabolism (Fig. 3c).

In contrast with the other two analyzed periods (maturation and adulthood), *Hnf1a* was not differentially expressed between ageing and middle-aged mice (Fig. 3d, *p*=*0.8002*), being also absent from the top upstream regulators (Supp. Fig. 1 g). Similarly, few β-cell markers were significantly deregulated in ageing mice (Fig. 3d). Moreover, there was no significant difference in the *Ins2, Sst or Ppy* gene expression levels (Supp. Fig. 1 h, *Ins2* [*p*=*0.3520*], *Sst* [*p*=*0.0776*], *Ppy* [*p*=*0.5762*]) or glycemia (Fig. 3e, *p*=*0.6195*) between the two age groups.

Interestingly, the insulin volume was significantly increased in ageing adults (Fig. 3 f, *p*=*0.0424*), while the Ins+ cells / islet section remained unchanged (Fig. 3 f, p=0.4063), suggesting an increase in volume (hypertrophy) but not in numbers of the beta-cell population. In contrast, the number of Sst+ cells / islet section (Fig. 3 g, *p*=*0.0204*) and Gcg+ cells / islet section (Fig. 3 h, *p*=*0.0495*) were significantly decreased. Similarly, *Gcg* was significantly downregulated *(−2.08x, p*=*0.0116*) in the 40-week-old mice, a trend also followed by other key alpha-cell markers, such as *Arx* (*p*=*0.0257*) and *Mafb (p*=*0.0199)* (Fig. 3i).

Taken together, the analysis of the ageing timeline indicates the presence of a regulatory inflection point for many metabolic functions and pathways in fully-grown adults, followed by the establishment of a steadily decaying molecular landscape in late adulthood (Fig. 3j, Supp Fig. 1 f). The data indicated Hnf1*a* as one of the main regulators of chronological ageing in the islet, however it is unclear which are the exact processes regulated by it.

### 3.3. Similar to HNF1A-MODY patients, suboptimal levels of Hnf1a impact critical metabolic processes at the onset of maturity

To address this issue and demultiplex the exact cellular and molecular functions regulated by Hnf1a during the age timeline, we generated a conditional mouse model allowing the knock-out of *Hnf1a* in a tissue specific manner (Supp. Fig. 2a). To study the effect of *Hnf1a* loss specifically in beta-cells, we crossed this model with a β-cell constitutive tagger (*RIP-Cre)* (Herrera et al., 1998). In this setup, beta-cells are efficiently recombined (96.21 %±6.3). In contrast to their human counterparts, adult mice heterozygous for the beta-cell specific *Hnf1a* mutation (Hnf1a^HTZ^) were normoglycemic, while the homozygous mutation (Hnf1a^HMZ^) caused mild hyperglycemia (~14 mmol/L) (Supp. Fig. 2b) and progressively impaired glucose tolerance. Yet, the weight gain was similar between the controls (RIP-Cre, Hnf1a^WT^), Hnf1a^HMZ^ and Hnf1a^HTZ^ animals (Supp. Fig. 2c). This is in line with the previously reported *Hnf1a* knock-out mouse models, which also required a homozygous mutation status to display a HNF1A-MODY-like phenotype (Lee et al., 1998; Pontoglio, 1996; Pontoglio, 1998; Parrizas, 2001).

To avoid the interference of hyperglycemia and diabetic phenotype on the metabolic functions and pathways readouts, we decided to use mice heterozygous for *Hnf1a* mutation. As expected, Hnf1a^HTZ^ mice exhibited suboptimal Hnf1a expression (Supp. Fig. 2d) as well as decreased levels of key beta-cell markers including insulin (Supp. Fig. 2e).

**Fig. 4.**
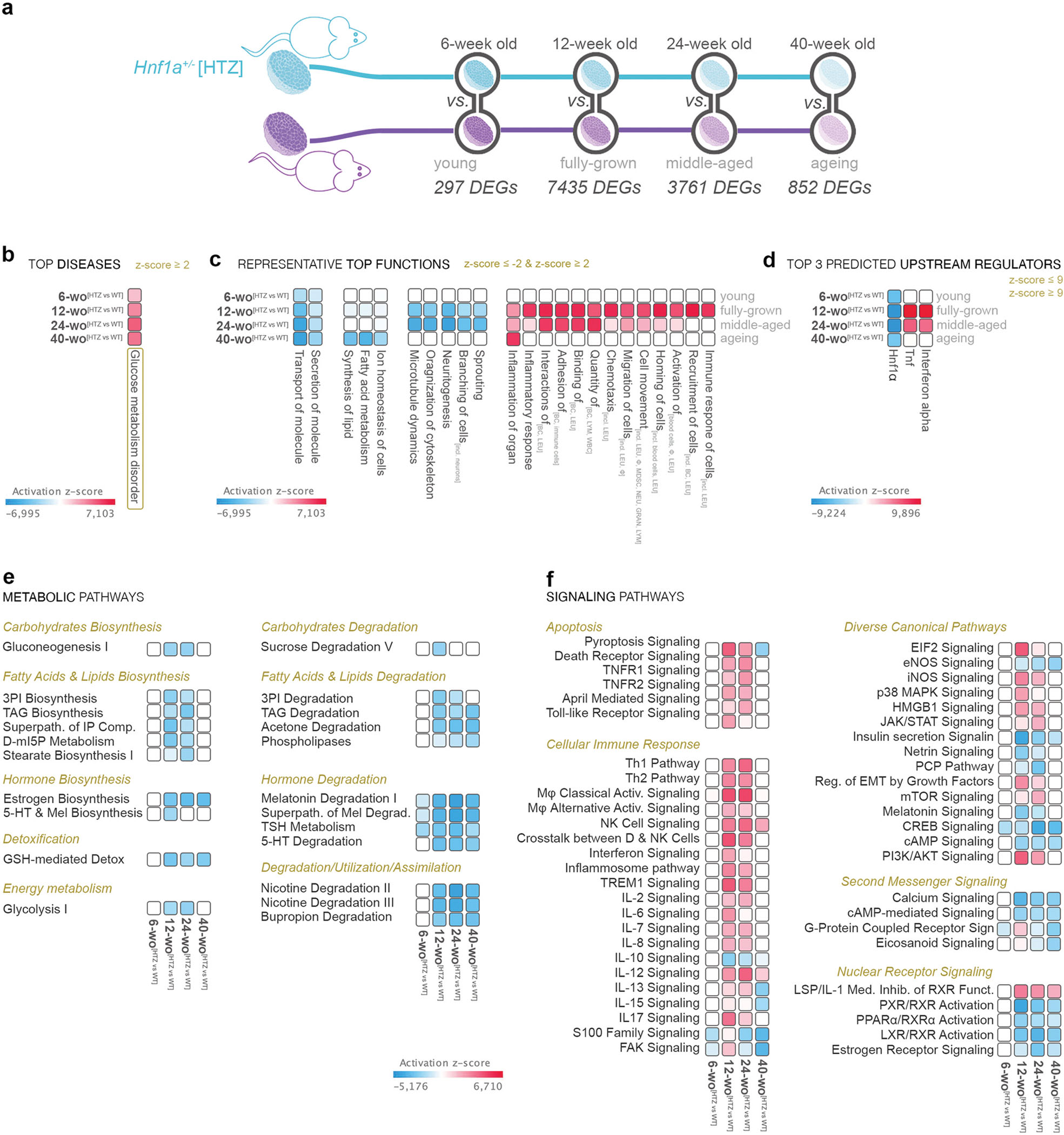
The pathway comparison of the age-specific impact of suboptimal *Hnf1a* levels. a) Scheme illustrating the compared ageing timelines with the four points of comparison analysis; b) The predicted activity pattern of glucose metabolism disorder amongst the four points of analysis (z-score ≥ 2 – activated – red); c) Comparison analysis of top representative functions, d) predicted upstream regulators, e) metabolic and f) signaling pathways amongst the four age stages (z-score ≥ 2 – activated – red; z-score ≤ −2 – inhibited - blue). (*p<0.05, **p<0.01, ***p<0.001, ****p<0.0001).

We thus performed transcriptomic analysis on isolated pancreatic islets from Hnf1a^HTZ^ mice at the same ages as before. The number of differentially expressed genes (FC≥1.5, p<0.05) between Hnf1a^HTZ^ and the age-matched controls varied in an age-specific manner, suggesting that suboptimal Hnf1a levels impact mostly the transcriptional landscape of the fully-grown (7435 DEGs) and middle-aged mice (3761 DEGs), while young mice are the least affected (297 DEGs) (Fig. 4a).

To identify the processes and pathways driven by these DEG sets, we performed a comparison analysis of all four time points. As expected, Glucose metabolism disorder was in the top Diseases and Disorders, being inferred increased across all age groups (Fig. 4b). Moreover, transport of molecule (top function) and secretion of molecule were predicted decreased at all time points (Fig. 4c), the only two functions with this pan-age regulatory dynamic. These data suggest that suboptimal Hnf1a levels affect glucose metabolism as well as molecular transport and secretion regardless of age.

In contrast, all other processes presented an age-specific activity pattern. For example, processes related to metabolism of lipid (such as fatty acid metabolism or lipid synthesis) and ion homeostasis were inferred as decreased particularly in ageing mice with suboptimal levels of Hnf1a (Fig. 4c). Of note, most functions had a change in the inferred activity pattern in both fully-grown and middle-aged mice, such as the decrease of processes involved in cytoskeleton regulation and the increase of those governing immune response and inflammation (Fig. 4c).

As anticipated, *Hnf1a* was the pan-landscape top upstream regulator (Fig. 4d). Of note, the top second and third were the proinflammatory cytokines *Tnf* and *interferon-alpha*, both being predicted activated in fully-grown and middle-aged, but not in young or ageing mice. Taken together these results suggest an activation of the immune response in fully-grown and middle-aged mice with suboptimal Hnf1a levels.

Furthermore, the comparison analysis of the canonical pathways revealed that signaling involved in carbohydrates biosynthesis (such as Gluconeogenesis), fatty acid & lipids biosynthesis (such as Triacylglycerols biosynthesis) and energy metabolism (such as Glycolysis) is predicted to be decreased in fully-grown and middle-aged Hnf1a^HTZ^ mice (Fig. 4e). In contrast, hormone metabolism (both biosynthesis and degradation), detoxification and lipid degradation were impacted by suboptimal Hnf1a levels either regardless of age or following the maturation stage.

Last, most pathways regulating diverse cellular and molecular processes were predicted deregulated in fully-grown and middle-aged Hnf1a^HTZ^ mice, including apoptosis (such as Death receptor signaling, TNFR1/TNFR2 signaling), cellular immune response (such as Th1 Pathway, Th2 Pathway, Interferon signaling, Inflammasome pathway and a wide variety of ILs Signaling) and developmental pathways (such as PCP, PI3K/AKT, Netrin pathways) (Fig. 4 f). Few others, such as the ones related to nuclear receptor signaling (such as Estrogen Receptor Signaling, LXR/RXR, PXR/RXR, PPAR*α*/RXR signaling), were inferred deregulated in mice with suboptimal levels of Hnf1a only after the onset of maturity.

Globally, these data suggest that similar to the human counterpart, suboptimal Hnf1a levels caused by the loss of one allele become evident with the onset of maturity. Despite remaining normoglycemic, the Hnf1a^HTZ^ presented an overall transcriptional landscape evocative of glucose metabolism disorder, with inactivation of pathways and processes involved in carbohydrate, lipid and energy, metabolism (especially in fully-grown mice) and an increase of the ones involved in immune response. Nevertheless, direct age comparison does not allow for an exact assessment of the process regulation dynamic over time and thus it is still unclear how suboptimal levels of Hnf1a impact maturation and ageing. Moreover, it must be established if there is an impaired/retained Hnf1a age-specific regulation or if this is abolished due to a decrease in Hnf1a levels.

### 3.4. Suboptimal Hnf1a level during maturation leads to a premature adulthood stage signature

**Fig. 5.**
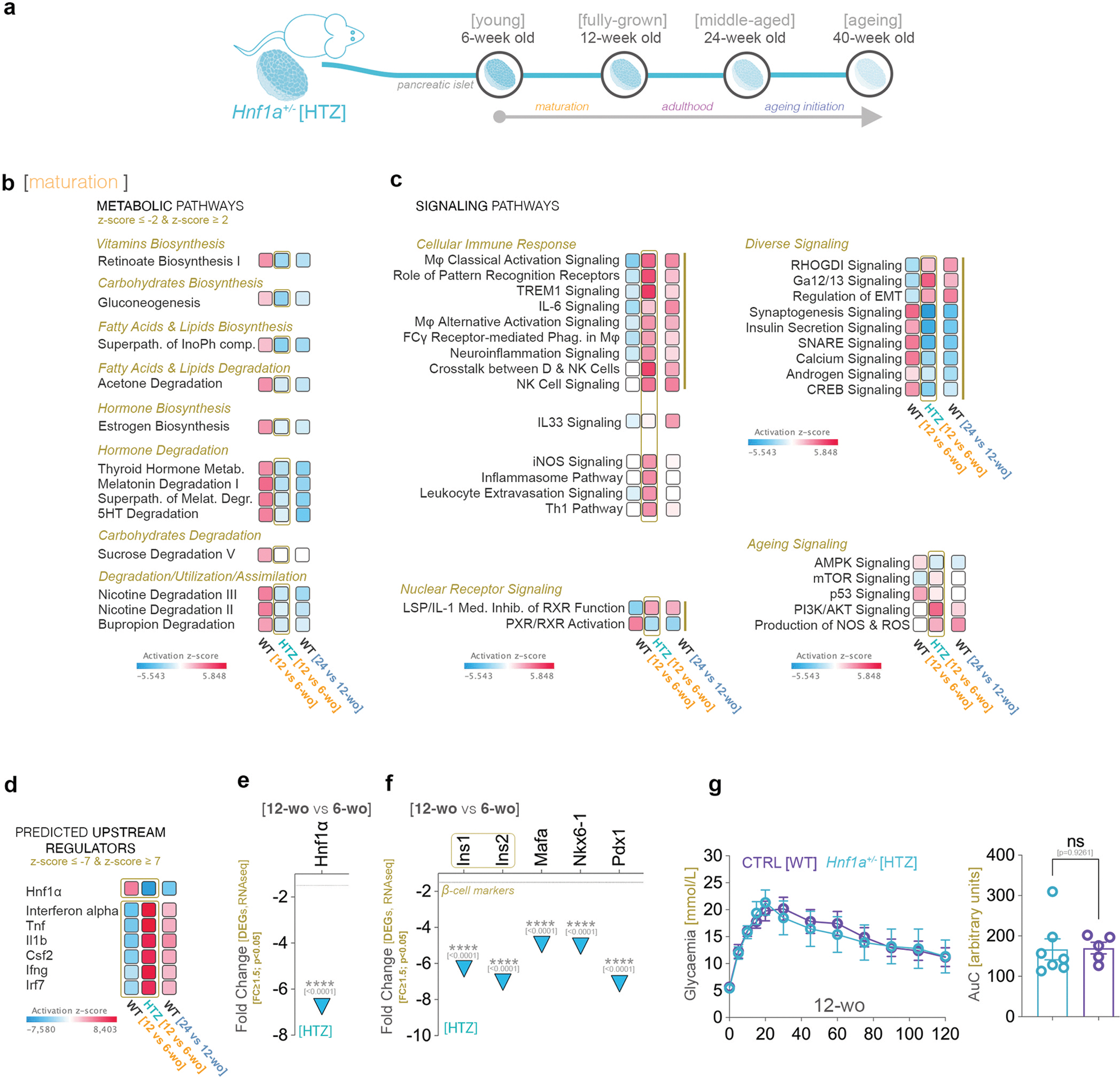
The age dynamic in mice with suboptimal *Hnf1a* levels. a) Scheme illustrating the timeline and the four points of analysis used in the *Hnf1*Hnf1*α* ^HTZ^ mice; b) Comparison analysis of metabolic and c) cellular immune response signaling pathways between the maturation and adulthood stages of control animals and the maturation stage of *Hnf1*Hnf1*α* ^HTZ^; d) Comparison analysis of predicted upstream regulators between the maturation and adulthood of control animals and the maturation stage of *Hnf1α* ^HTZ^ (z-score ≥ 7 – activated – red; z-score ≤ −7 – inhibited - blue). e) Graph displaying the observed downregulation of *Hnf1a* during the maturation stage in *Hnf1α* ^HTZ^ (FC ≥ 1.5, p < 0.05); f) Graph displaying the observed downregulation selected differentially expressed beta-cell markers, including the insulin genes, based on RNAseq data (FC ≥ 1.5, p < 0.05); g) Graph depicting the IPGTT dynamic (intra-peritoneal glucose tolerance test) and associated area under curve (AUC) comparing the glucose tolerance of the control and *Hnf1*Hnf1*α* ^HTZ^ mice in fully-grown (12-week-old) animals (non-parametric Mann-Whitney test, each data point represents one distinct islet section, each data point represents one distinct animal). (*p<0.05, **p<0.01, ***p<0.001, ****p<0.0001).

To map the chronology of the molecular processes characterizing the ageing timeline in mice with suboptimal levels of Hnf1a, we analyzed the same developmental periods along the Hnf1a^HTZ^ timeline (Fig. 5a).

The comparative pathway analysis revealed a stark regulatory contrast between the Hnf1a^HTZ^ mice maturation period and the one of Hnf1a^WT^ mice (Fig. 5b, c, compare first and second column), the activity profile during this period being reminiscent of the one presented during adulthood in WT mice (Fig. 5b, c, compare second column and third column).

The activity pattern of most metabolic and signaling pathways in mice maturing under suboptimal Hnf1a levels (Hnf1a^HTZ^) was opposite to the one observed during normal maturation phase, evocating the one observed during the adulthood period in Hnf1a^WT^ mice (Fig. 5b, c). Amongst these, there were pathways involved in degradation of lipids and certain hormones, as well as biosynthesis of vitamins (retinoate) and hormones (estrogen) (Fig. 5b). In addition, the activity pattern of many developmental signaling, such as the ones involved in the cellular immune response, nuclear receptor signaling or pathways with known role in ageing progression (such as AMPK, mTOR, PI3K/AKT and ROS & NOS signaling) followed the same inverted trend (Fig. 5c), i.e. opposing the one observed during normal maturation and reminiscent to the one inferred during the adulthood stage in Hnf1a^WT^ mice (Fig. 5c).

In contrast, a second category of metabolic pathways, involved in the degradation of lipids and hormones, were unchanged between young and fully-grown Hnf1a^HTZ^ mice, despite being regulated during the maturation and adulthood in healthy mice with optimal Hnf1a levels (Supp. Fig. 3a). Moreover, a minority of pathways (such as Triacylglycerol (TAG) Degradation) were inferred regulated only during maturation in suboptimal Hnf1a levels (Supp. Fig. 3b). The few exceptions retaining the same activity pattern observed in mice with normal Hnf1a levels were involved in amino acid metabolism (Methionine, Valine and Cysteine) and electron transfer (Supp. Fig. 3c).

Correspondingly, the top predicted upstream regulators retained an opposing activity pattern between maturing Hnf1a^WT^ and Hnf1a^HTZ^ mice. Moreover, the Hnf1a inferred activity pattern was always in antithesis with the ones exhibited by the anti-inflammatory cytokines presented in the top (such as *Tnf, Il1b* and *Ifng*), regardless of the condition (Fig. 5d). Of note, *Hnf1a* was strongly predicted as inactivated during this stage in Hnf1a^HTZ^ mice (activation z-score −7.580). Indeed, in suboptimal Hnf1a level conditions, *Hnf1a* was observed downregulated in fully-grown as compared to young mice (Figs. 5e, *−6.759x p*<*0.0001*), this being in stark contrast to the upregulation observed during the normal maturation stage (compare with Fig. 1 f). As expected, key beta cell markers (Fig. 5 f), including the insulin genes, were also downregulated. Of interest, except for *Ins1*, *Ins2*, these genes presented an opposite regulatory pattern (upregulated) during the normal maturation stage (see Fig. 1 g). Despite this decrease in the expression level, there was no significant difference in the glucose tolerance between Hnf1a^WT^ and Hnf1a^HTZ^ fully-grown adults (Fig. 5 g, *p*=*0.9261*) measured by ipGTT, indicating the presence of compensatory mechanisms.

Furthermore, the pathway analysis of the adulthood stage in Hnf1a^HTZ^ mice revealed a swap in the activity pattern of several metabolic pathways (involved in lipid metabolism and pathway transport) compared to the previous stage (Supp Fig. 3d), indicating the maintenance of the regulatory inflection point in fully-grown Hnf1a^HTZ^ adults, although with an opposite regulation pattern than in Hnf1a^WT^. This was also true for immune system signaling, which was inferred decreased at this stage (Supp Fig. 3e) and was accompanied by an upregulation of the Hnf1a expression levels (Supp Fig. 3 f).

Globally, these data suggest that mice growing under suboptimal Hnf1a levels are unable to deploy the normal maturation program, presenting a regulatory activity evoking the adulthood patterns, such as the activation of pathways involved in the immune system response. Moreover, the fact that, besides its already suboptimal levels, there is also an age-specific Hnf1a deregulation, suggests the presence of a regulatory loop, in which targets affected by Hnf1a reduced levels, regulate, in turn, its activity. Of interest, the regulatory pattern of the immune response (especially lymphocyte and NK signaling) invariably opposed the one of Hnf1a during both postnatal development and genetic alteration, indicating a possible connection between the two.

### 3.5. Hnf1a age-specific regulation is replaced by a sustained expression in immunodeficient mice

To further explore the link between *Hnf1a* and immune response, we investigated the pattern of Hnf1a regulation and the chronology of molecular processes during adult development progression in NSG (*NOD scid gamma*) immunodeficient mice. The NSG animals are extremely immunodeficient, carrying two distinct mutations (i) *scid* (*Prkdc* mutation) and *IL2rg*^*null*^ (complete null allele of IL2 receptor gamma chain) on a NOD genetic background. These two mutations render the mice deficient in mature T and B lymphocytes (*scid*), while NK cells cytotoxic activity is very low (*IL2rg*^*nul*^*)*. Consequently, in contrast with NOD mice, NSG mice do not develop spontaneous diabetes.

**Fig. 6.**
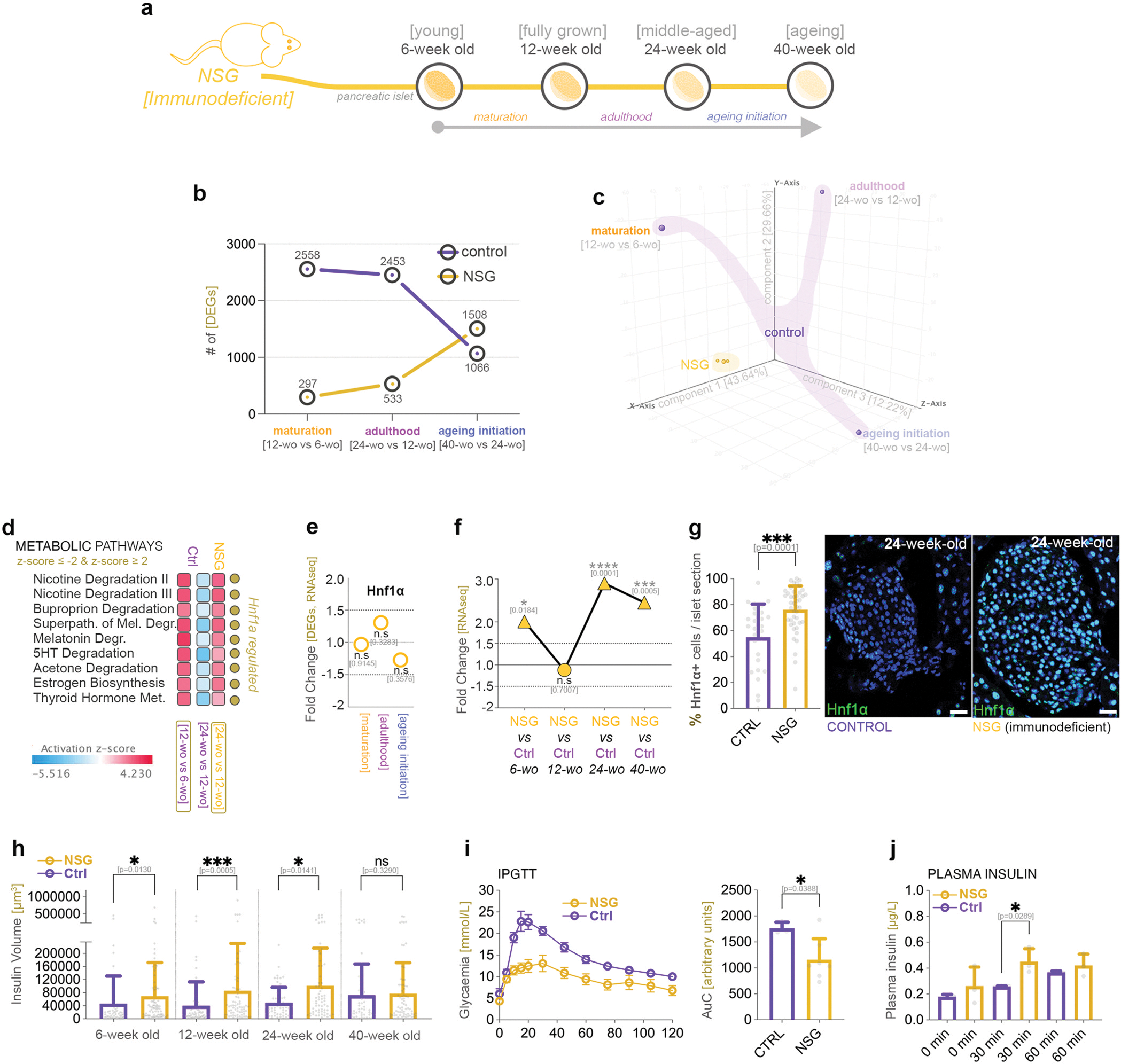
The age dynamic in immunodeficient mice. a) Scheme illustrating the ageing timeline and the four points of analysis used in the *NSG* immunodeficient mice; b) Graph depicting the number of differentially expressed genes (DEGs, FC ≥ 1.5, p < 0.05) during maturation, adulthood and ageing initiation in NSG and immunocompetent control mice; c) 3D PCA plot of the NSG and immunocompetent control stages (3 components); d) Comparison analysis of the metabolic pathways between the immunocompetent control mice and immunodeficient NSG animals (z-score ≥ 2 – activated – red; z-score ≤ −2 – inhibited - blue); e) Graphs displaying the observed non-significant regulation of *Hnf1α* in the RNAseq dataset during the maturation, adulthood and ageing initiation in NSG mice; f) Graph depicting the differential expression of *Hnf1α* (FC ≥ 1.5, p < 0.05) between NSG and control immunocompetent mice; g) Graph depicting the manual quantification of the number of Hnf1*α* positive cells per islet section (non-parametric Mann-Whitney test, each data point represents one distinct islet section, an average of 12 islets were assessed / mouse; and 3 mice were analyzed per condition) and representative immunofluorescence images of Hnf1*α* staining in NSG and control middle-aged adults (scale – 20 μm, green – Hnf1*α*); h) Direct comparison of insulin volume between NSG and control mice along the timeline (non-parametric Mann-Whitney test, each data point represents one distinct animal); i) IPGTT dynamic and corresponding AUC comparing glucose tolerance between the control and *NSG* mice (non-parametric Mann-Whitney test, each data point represents one distinct islet section, each data point represents one distinct animal); j) Graph depicting the plasma insulin in control and *NSG* mice (unpaired t-test with Welch’s correction, each data point represents one distinct animal) (*p<0.05, **p<0.01, ***p<0.001, ****p<0.0001).

As before, we performed the transcriptomic analysis on isolated pancreatic islets from NSG mice at the same ages as previously considered (Fig. 6a). Along the ageing timeline fewer deregulated genes were detected in immunodeficient mice than in the immunocompetent controls (Fig. 6b), with the larges difference being observed during maturation (297 vs 2558 DEGs), suggesting that the normal maturation program is impacted by the absence of critical immune system components. Furthermore, the PCA analysis clearly discriminated between normal and immunodeficient groups (Fig. 6c). In contrast, the separation by life stage only occurred for the immunocompetent, but not NSG group, which were still clustered together, indicating fewer transcriptional differences between the stages in immunocompromised mice.

In the NSG mice, the comparison pathway analysis inferred the presence of an activity pattern for all the metabolic pathways observed to be deregulated in an age-specific manner by the suboptimal levels of Hnf1a (Fig. 6d, Fig. 5b). Of note, their activity profile is similar to the one presented during normal maturation in control immunocompetent mice. Interestingly, while this is reversed during the next stage in normal healthy mice, it is initiated during the adulthood of the immunodeficient counterpart (Fig. 6d).

In contrast, pathways which were not impacted by Hnf1a levels, such as the ones involved in amino acids metabolism, followed a different activity pattern, being largely not regulated in NSG mice until ageing initiation (Supp. Fig. 4a).

Considering that during normal maturation the inferred activity status of the pathways regulated by Hnf1a was in direct relationship with the *Hnf1a* expression levels, we investigated the *Hnf1a* expression pattern. This was unchanged during all stages (Fig. 6e), suggesting that *Hnf1a* modulations are dependent on an immunocompetent environment. Moreover, neither the three main islet hormones (Supp. Fig. 4b), nor key beta- or alpha-cell specific markers (Supp. Fig. 4c) were found deregulated along the timeline.

To identify if the lack of *Hnf1a* age-specific regulation is caused by constantly high levels or, alternatively, by a transcriptional block, we compared *Hnf1a* expression between the immunodeficient and immunocompetent environments. *Hnf1a* was found to be significantly upregulated in the NSG mice (Fig. 6 f) at all time points except in fully-grown adults (12-week-old), the reason for this exception being the transient *Hnf1a* upregulation at this particular time point in immunocompetent mice (see Fig. 1 f). Confirmatory, we also observed more cells expressing Hnf1a protein in NSG islets (Fig. 6 g). These data suggest that in the absence of a competent immune system the transient regulation of *Hnf1a* observed during normal postnatal development is replaced by a sustained expression, possibly explaining the activation pattern inferred for the metabolic pathways regulated by *Hnf1a*.

Consistent with the *Hnf1a* expression pattern, key beta-cell markers exhibited the same sustained upregulation trend (Supp. Fig. 4d). Furthermore, the insulin volume was significantly higher in immunodeficient mice at all time points except ageing 40 weeks-old adults (Fig. 6 h), suggesting that constantly increased *Hnf1a* levels might lead to changes in islet size and functionality. Indeed, glucose tolerance was improved in NSG mice (showed by ipGTT in Fig. 6i, *p*=*0.0388*), probably due to an increased insulin release at 30 minutes following glucose administration (see plasma insulin levels in Fig. 6j, *p*=*0289*).

Overall, these data suggest that, along with the anticipated immune system signature differences, the immunodeficient mice with sustained *Hnf1a* levels present age-specific metabolic changes.

### 3.6. HNF1A regulation and metabolic pathways profile are similar between human and mice

To establish if any of the above observations are translatable in human islets, we further assessed human islets isolated from cadaveric donors following their grouping according to the age decade. Due to the scarcity of the material and limitations related to the pre-processing of the material, we could analyze only three age-groups (i) 35–49 – (here after quadragenerians or 40 s), (ii) 50–59 (here after quinquagenarians 50 s) and (iii) 60–69 (here after sexagenarians or 60 s) by TMT-plex global proteomics.

**Fig. 7.**
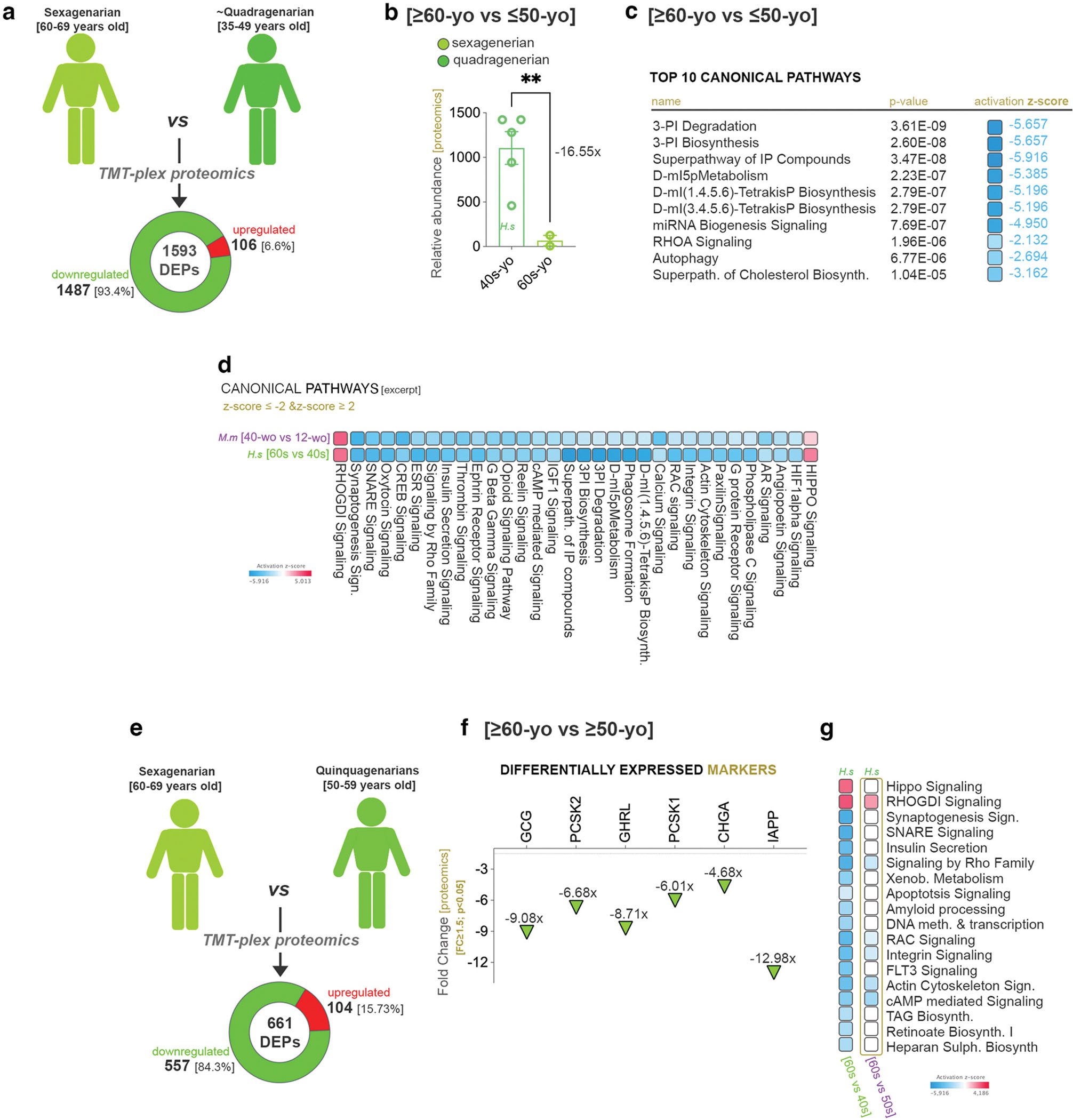
TMT-plex proteomics comparison between human islets of different age groups. a) Scheme illustrating the experimental setup and the number of proteins with different abundance (DEPs) between sexagenarian and quadragenerian human islets (FC ≥ 1.5, p < 0.05). b) Graph of the decreased Hnf1a protein abundancy in sexagenarians (each data point represents one distinct donor islet). c) Top 10 canonical pathways of the analyzed differential islet proteome landscape between sexagenarians and quadragenerians. d) Comparison pathway analysis between the differential landscapes characterizing the sexagenarians and mice. e) Scheme illustrating the experimental setup and the number of proteins with different abundance (DEPs) between sexagenarian and quinquagenarian human islets (FC ≥ 1.5, p < 0.05). f) Graph of key hormone and islet cell markers with decreased protein abundancy levels in sexagenarians. g) Comparison pathway analysis between the differential proteome landscapes of the two islet age groups’ comparisons (60 s vs 40 s and 60 s vs 50 s). (*p<0.05, **p<0.01, ***p<0.001, ****p<0.0001).

We first performed the comparison between quadragenerians and sexagenarians, which revealed 1583 proteins with significantly different (FC≥1.5, p<0.05) abundancy levels (DEPs) in islets (Fig. 7a). Interestingly, very few proteins presented increased abundancy in sexagenarians (106), most being exhibiting decreased levels (93.4 %). Importantly, HNF1A was detected amongst these DEPs, being downregulated (−16.55x) in the older group (Fig. 7b), suggesting its involvement in the regulation of age-specific processes also in humans.

The pathway analysis revealed signaling involved regulating the carbohydrate and lipid metabolism balance, second messenger signaling and autophagy (Fig. 7c), all inferred as inhibited in the sexagenarians. Furthermore, the comparison analysis revealed several canonical pathways, which exhibited the same activity pattern as the one inferred between ageing and fully-grown mice (Fig. 7d). Amongst these, there were pathways involved in lipid metabolism, growth (such as Hippo signaling, Autophagy, Insulin Secretion, SNARE) and intracellular signaling (such as RAC, RHOGDI, Integrin). Of interest, only two pathways were inferred as activated: Hippo (involved in regulating organ size, growth and proliferation) and RHOGDI (cytoskeletal organization, membrane trafficking, growth).

Last, the comparison between quinquagenarians’ and sexagenarians’ islets revealed only 661 DEPs (Fig. 7e). As before, most proteins exhibited significantly less abundancy levels in the older group (84.3 %). Interestingly almost the same number of proteins were detected to be more abundant in sexagenarians (104 DEPs), with 50 (~50 %) being commonly upregulated in both comparisons. In this comparison HNF1A was not significantly regulated (p=0.063), however several key endocrine (CHGA) and islet markers, including the hormones GCG and Ghrelin (GHRL, detectable only in the adult human, but not mouse, islet), showed a significant decrease in abundancy levels (Fig. 7 f). Of interest, the comparison analysis determined that most of the pathways with a predicted activity pattern in the previous comparison (between the more distant groups the sexagenarians and quadragenerians) were not inferred regulated between the two older groups (Fig. 7 g, compare first and last column).

Overall, these data suggest a certain level of conservation of the age-specific regulatory landscapes and metabolic processes between mice and human islets.

## 4. Discussions

The complexity of the maturation and ageing processes, involving a wide range of cellular and molecular changes, makes their comprehensive characterization extremely challenging. In this study we focused on the characterization of the dynamic transcriptional changes characterizing the murine islet adult development process. Briefly, we show that post-pubertal murine islets exhibit significant non-linear changes in the transcriptional landscape during maturation, adulthood, and ageing initiation. Their developmental curve is characterized by a regulatory inflection point in fully-grown adults (~12-week-old). We identified that Hnf1a, a key regulator of pancreatic islet fate and functionality, follows the same regulatory dynamic. Suboptimal levels of *Hnf1a*, caused by the loss of one allele, reverse its age-specific regulatory dynamic, impacting mostly the maturation stage. This leads to a switch in the activity pattern of several key metabolic processes, generating a regulatory landscape similar to the one observed during the adulthood of control mice. In addition, the regulatory pattern of the immune response signaling invariably opposed the one of Hnf1a during both postnatal development and genetic alteration. We observe that in severely immunodeficient mice, Hnf1a age-specific regulation is replaced by sustained expression coupled with improved glucose tolerance and age-specific metabolic changes. Finally, we show that the regulatory profile of HNF1A as well as the one of many signaling and metabolic pathways was similar in adult human islets.

Changes in energy homeostasis represent one of the main hallmarks of adulthood progression and ageing. Lipid metabolism plays an important role in life-span regulation (Johnson and Stolzing, 2019), with the ability of oxidizing fat being shown to decrease with age (Toth and Tchernof, 2000). Moreover, studies in several model systems, including rodents, demonstrated that different genetic and nongenetic interventions leading to improved lipid metabolism significantly extend lifespan and is accompanied by enhanced tolerance to oxidative stress (Johnson and Stolzing, 2019). During the final maturation stage (from young to fully-grown adults), we identified a switch in the energy balance, defined by an increase in lipid- and a decrease in carbohydrate metabolism, coupled with reduced ROS synthesis. Interestingly, as lipid signaling is absolutely necessary for the *in vivo* beta-cells’ glucose- and non-glucose-stimulated insulin secretion (Dobbins, 1998; Langlois et al., 2021; Roduit, 2004), these results suggest a more effective islet function in fully-grown adults and an improved energetic profile. This is accordance with previous studies (Stolovich-Rain, 2015) indicating that weaning (i.e. switch from milk-based diet to standardized chow usually around 3-weeks of age) triggers a discrete maturation step in the islet. Admittedly, our first analysis time-point (6-week-old) is significantly later than the moment of weaning (3-week-old), suggesting that the maturation process still refines between 6- and 12-weeks of age.

In contrast, in middle-aged adults, lipid oxidation (especially fatty acid) is decreased, while ROS synthesis is increased, a combination observed in many tissues during the normal ageing process (Johnson and Stolzing, 2019; Levadoux, 2001; Solomon et al., 2008). Moreover, the age-dependent modulation of energy metabolism was also reported by *in vivo* quantitative proteomics following a comparison in 1-month-old and 1-year-old mice (Wortham, 2018). However, the wide time range covered by this comparison and the difference in time-points does not allow for a comparison analysis with the data presented here.

Another feature of ageing is immunosenescence leading to chronic low-grade inflammation caused by the persistent activation of the immune system (Fulop, 2017; Franceschi, 2000). Indeed, the transient downregulation in the inflammatory response observed in fully-grown mice, was reversed during the later stages. This is in line with previous observations in rat islets, where an age-related persistent low-grade inflammation status was identified by comparing two age time points) (Sandovici, 2016).

The age-dependent hormone regulation pattern was characterized by a significant reduction of somatostatin and glucagon expression with adulthood progression. The decrease of glucagon expression with age was previously reported in an older study, while the same work did not observe a decrease in somatostatin, probably because of the difference in the sensitivity of the technique employed (Perfetti et al., 1996).

At cellular level, the decrease in hormone expression was paralleled by a significant decrease in glucagon and somatostatin cell numbers in ageing adults. In contrast, the insulin volume increased, while insulin transcripts remained stable. This apparent counterintuitive tendency is resolved by an elegant study by Dor and colleagues (Anzi, 2018) showing that mouse beta cells grow significantly during postnatal life, reaching a peak in cell volume at around 8.5 mo (~34-week-old). These observations were also confirmed by independent studies addressing the morphology and physiology of ageing islets, showing an aged-related increase in islet size and beta cell mass (Tuduri, 2022), while glycemia and insulin levels in the plasma were unchanged with age even in very old mice (Kehm, 2018). If this is a compensatory mechanism for coping with the increase in insulin demand or age-related decline in beta-cell function remains to be uncovered (Anzi, 2018). Although surprising, the stable insulin expression along the age timeline was also observed in rat islets by qPCR quantification in rats from to 2- to 24-month-old (Giddings et al., 1995) as well as in human islets of diverse ages, where insulin mRNA levels were also stable with age (Arda, 2016).

Pathway and network analysis of the transcriptomic landscape proved to be an efficient tool for pinpointing novel gene candidates regulating the ageing process (Kaletsky, 2018; Wang, 2022; Yang, 2016; Cohen, 2022). By using this approach, we identified Hnf1a as a top upstream transcriptional regulator with observed modulations during maturation, adulthood, and ageing initiation. Hnf1a is a key regulator of pancreas development (Servitja, 2009), its suboptimal expression levels being responsible for the most prevalent form of maturity onset monogenic diabetes (HNF1A-MODY or MODY3) (Yamagata, 1996). Here we used a novel conditional mouse model that allowed the study of the post-natal development in the pancreatic islet with suboptimal levels of Hnf1a, however in the absence of the confounding effects caused by hyperglycemia and diabetes. Interestingly, similar to the human counterpart, the effect of suboptimal Hnf1a levels on the transcriptional landscape become evident with the onset of maturity. Of note, the maturation stage regulatory fingerprint in these mice resembles the one mapped during healthy control adulthood, characterized by reduced lipid oxidation and increased inflammation, suggesting a possible premature metabolic maturation in this model. Moreover, the activity pattern of the immune response (especially lymphocyte and NK signaling) invariably opposed the one of Hnf1a during both maturation and genetic alteration, indicating a possible connection between the two.

To our knowledge, this is the first study involving Hnf1a in the natural maturation/ageing processes, besides a recent report linking, in silica, Hnf1a to the low muscle mass in elderly women (Jales Neto, 2021). In contrast, there is a slightly larger body of recent literature linking Hnf1a to inflammation and immune system response in diverse disease models, such as heart disease or steatohepatitis (He, 2022; Armendariz and Krauss, 2009). However, the finding of a direct or indirect regulatory loop between Hnf1a and the immune system signaling, especially inflammation is, to our knowledge, new. We show here that suboptimal Hnf1a levels increased the inflammatory activity, while in immunodeficient mice Hnf1a expression is persistently upregulated as compared to immunocompetent mice. This suggests a regulatory crosstalk, yet it also stems a chicken and egg dilemma regarding the trigger of this loop, which will require further investigation. Based on the principle of Occam’s razor (“The simplest explanation is the most likely one”) it is tempting to speculate that the chronic low-grade inflammation occurring with islet chronologically ageing passively causes the decrease of Hnf1a expression, a scenario backed by Hnf1a constitutively high levels in immunodeficient mice. In contrast, Hnf1a actively modulates the immune system signature as its suboptimal level triggers a regulation pattern inversion of both immune signaling and itself.

Taken together, we propose here a model where Hnf1a is involved in islet post-natal development by regulating the energy metabolism and immune signaling, being in turn controlled by immune system modulations.

Nevertheless, while endorsing this model, one should carefully consider the specific limitations of the study, like the absence of a comprehensive experimental validation of the observed immune signatures. Moreover, the important impact of the inherent mouse strain differences on the observed differential signature between the immunodeficient and immunocompetent backgrounds should be assumed, however this could not be demultiplexed in the current analyses. Along the same line, this study is almost entirely based transcriptomics analysis, thus it is not covering the posttranscriptional and posttranslational modifications that could impact the actual process regulation. Despite these limitations, the results presented here aim to promote future experimentally driven research into further deciphering the Hnf1a role in ageing and inflammation.

## Supplementary Material

Table S2

Table S1

Table S3

Table S4

Figure S1

Figure S2

Figure S3

Figure S4

## Data Availability

Data will be made available on request.

## Appendix A. Supporting information

Supplementary data associated with this article can be found in the online version at doi:10.1016/j.mad.2024.111951.
