# Supplementary material for: The age-dependent regulation of pancreatic islet landscape is fueled by a HNF1a-immune signaling loop
Source: Mech Ageing Dev. Author manuscript; Available in PMC 2025 Dec 16. (PMC12706456; doi:10.1016/j.mad.2024.111951)
Supplement: Table S2 [file NIHMS2123777-supplement-Table_S2.pdf]

Table S1. Checklist for reporting human islet preparations used in research

Adapted from Hart NJ, Powers AC (2018) Progress, challenges, and suggestions for using human islets to understand islet biology and human diabetes. Diabetologia https://doi.org/10.1007/s00125-018-4772-2

**Islet preparation**	**1**	**2**	**3**	**4**	**5**	**6**	**7**	**8[a]**
**MANDATORY INFORMATION**								
Donor age (years)	48	60	49	35	59	57	46	57
Donor sex (M/F)	M	M	F	M	M	M	M	F
Donor BMI (kg/m^2)	27.8	27.8	23.5	25.2	22.5	234.5	29.2	24.2
Donor HbA_{1c} or other measure of blood glucose control	33	44	34	NA	NA	NA	NA	NA
Origin/source of islets[b]	ECIT	EDIT	ECIT	ECIT	ECIT	ECIT	ECIT	ECIT
Islet isolation centre	Oslo	Oslo	Oslo	Oslo	Oslo	Oslo	Oslo	Oslo
Donor history of diabetes? Please select yes/no from drop down list	No	No	No	No	No	No	No	No
**If Yes, complete the next two lines if this information is available**								
Diabetes duration (years)								
Glucose-lowering therapy at time of death[c]								
**RECOMMENDED INFORMATION**								
Donor cause of death	DBD	DBD	DBD	DBD	DBD	DBD	DBD	DBD
Warm ischaemia time (h)	02:00	02:03	01:40	03:01	02:46	01:44	02:12	02:25
Cold ischaemia time (h)	03:02	02:30	08:00	08:30	05:29	02:19	01:00	05:49

Estimated purity (%)	53	50	70	68	60	67	45	60
Estimated viability (%)	90	80	90	95	95	87	90	90
Total culture time (h)[d]	72	72	72	72	72	72	72	72
Glucose-stimulated insulin secretion or other functional measurement[e]	2.4	2.3	4.3	2.1	2.5	4.0	3.5	2.3
Handpicked to purity? Please select yes/no from drop down list	Yes	Yes	Yes	Yes	Yes	Yes	Yes	Yes
Additional notes								

[a]If you have used more than eight islet preparations, please complete additional forms as necessary
[b]For example, IIDP, ECIT, Alberta IsletCore
[c]Please specify the therapy/therapies
[d]Time of islet culture at the isolation centre, during shipment and at the receiving laboratory
[e]Please specify the test and the results

Table S1. Checklist for reporting human islet preparations used in research (continuation)

Adapted from Hart NJ, Powers AC (2018) Progress, challenges, and suggestions for using human islets to understand islet biology and human diabetes. Diabetologia https://doi.org/10.1007/s00125-018-4772-2

**Islet preparation**	**1**	**2**	**3**	**4**	**5**	**6**	**7**	**8[a]**
**MANDATORY INFORMATION**								
Donor age (years)	36	67	49	52				
Donor sex (M/F)	F	M	F	F				
Donor BMI (kg/m^2)	20.8	26.9	17.0	34.9				
Donor HbA_{1c} or other measure of blood glucose control	NA	NA	NA	NA				
Origin/source of islets[b]	ECIT	EDIT	ECIT	ECIT				
Islet isolation centre	Oslo	Oslo	Oslo	Oslo				
Donor history of diabetes? Please select yes/no from drop down list	No	No	No	No				
**If Yes, complete the next two lines if this information is available**								
Diabetes duration (years)								
Glucose-lowering therapy at time of death[c]								
**RECOMMENDED INFORMATION**								
Donor cause of death	DBD	DBD	DBD	DBD				
Warm ischaemia time (h)	02:50	03:13	03:18	01:00				
Cold ischaemia time (h)	03:02	08:00	01:00	08:30				

Estimated purity (%)	53	50	70	68				
Estimated viability (%)	90	80	90	95				
Total culture time (h)[d]	72	72	72	72				
Glucose-stimulated insulin secretion or other functional measurement[e]	2.4	2.3	2.2	2.4				
Handpicked to purity? Please select yes/no from drop down list	Yes	Yes	Yes	Yes				
Additional notes								

[a]If you have used more than eight islet preparations, please complete additional forms as necessary
[b]For example, IIDP, ECIT, Alberta IsletCore
[c]Please specify the therapy/therapies
[d]Time of islet culture at the isolation centre, during shipment and at the receiving laboratory
[e]Please specify the test and the results
