# Supplementary material for: The age-dependent regulation of pancreatic islet landscape is fueled by a HNF1a-immune signaling loop
Source: Mech Ageing Dev. Author manuscript; Available in PMC 2025 Dec 16. (PMC12706456; doi:10.1016/j.mad.2024.111951)
Supplement: Table S1 [file NIHMS2123777-supplement-Table_S1.docx]

SUPPLEMENTARY FIGURES LEGENDS:

Supplemental Figure 1. **Normal Chronological Timeline**

**a)** Top Molecular and Cellular Functions characterizing the normal maturation period (12-week-old vs 6-week-old). **b)** Molecular functions with predicted activation pattern characterizing the normal maturation period (z-score ≥ 2 – activated; z-score ≤ -2 – inhibited). **c)** Graph displaying the observed regulation of the main islet hormones during characterizing the normal maturation period. **d)** Representative confocal images of insulin staining (red – insulin; blue – DAPI; scale – 100 µm) in 6- and 12-week-old mice **e)** The activity pattern of metabolic functions characterizing the ageing initiation period. **f)** Comparison analysis of the molecular and cellular functions activity pattern between the different stages (z-score ≥ 2 – activated – red; z-score ≤ -2 – inhibited - blue); **g)** Top predicted upstream transcription regulators with observed regulation during the normal ageing initiation stage. **h)** Graph displaying the observed regulation of *Ins2*, *Sst* and *Ppy* during the normal ageing initiation.

Supplemental Figure 2. **The Hn1α Mouse Model**

**a)** The Hnf1**α **mouse model design strategy. **b)** Evolution of glycemia in mice homozygous and heterozygous for *Hnf1a* defloxing (18 mice / condition) and their specific control (12 mice). **c)** Weight gain in mice homozygous and heterozygous for *Hnf1a* defloxing and their specific control (12 mice / condition). **d)** Graph displaying the observed downregulation (FC ≥ 1.5, p < 0.05) of *Hnf1α* in *Hnf1a*^HTZ^ mice at 12 weeks of age as revealed by RNAseq. **e)** Graph depicting the observed downregulation of selected differentially expressed (FC ≥ 1.5, p < 0.05) beta-cell markers in *Hnf1a^[HTZ^*^]^ mice as revealed by RNAseq.

Supplemental Figure 3. **Activity patterns during Hnf1α^HTZ^ postnatal development timeline**

**a-c)** Different pathway activity patterns revealed by comparison pathway analysis between the maturation and adulthood stage of control animals and the maturation stage of *Hnf1α*^HTZ^; **d)** Metabolic pathways and **e)** immune signaling comparison analysis between maturation and adulthood stage in the *Hnf1α*^HTZ^; **f)** Graph displaying the observed significant upregulation of *Hnf1a* in Hnf1α^HTZ^ mice during adulthood (FC ≥ 1.5, p < 0.05);

Supplemental Figure 4. **Timeline in the immunodeficient NSG model**

**a)** The activity pattern of pathways involved in amino acids metabolism; **b,c)** Graphs displaying the observed non-significant regulation in the RNAseq dataset of the main islet hormones **(b)** and key beta- and alpha-cell markers **(c)** along the timeline in the immunodeficient NSG model. **d)** Graph depicting the differential expression (FC ≥ 1.5, p < 0.05) of beta-cell specific markers between NSG (immunodeficient) and matched controls (immunocompetent) along the timeline (6-, 12-, 24- and 40-week-old) as revealed by RNAseq.
