# Supplementary figures and images for: The age-dependent regulation of pancreatic islet landscape is fueled by a HNF1a-immune signaling loop
Source: Mech Ageing Dev. Author manuscript; Available in PMC 2025 Dec 16. (PMC12706456; doi:10.1016/j.mad.2024.111951)

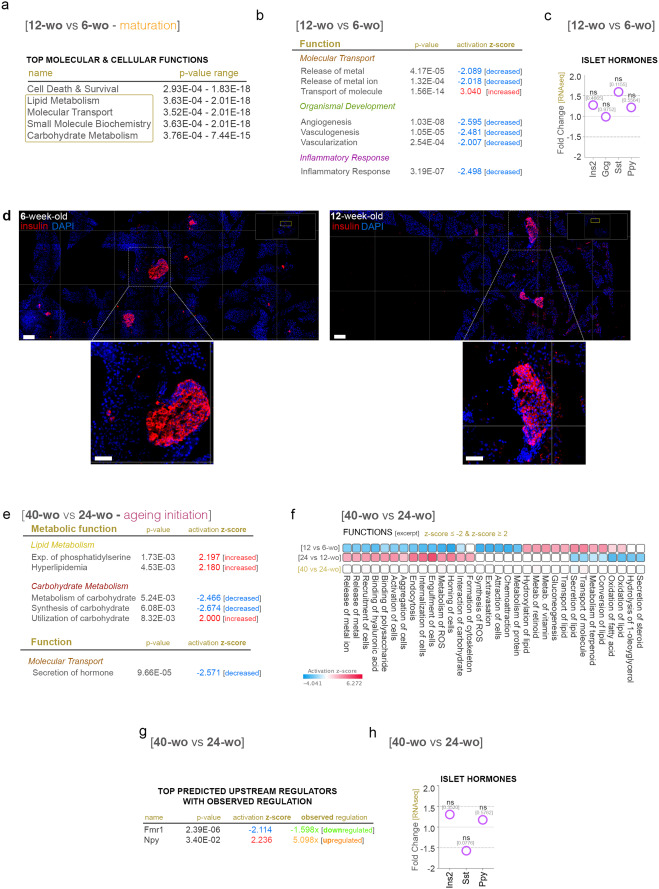

Supplement: Figure S1 [file NIHMS2123777-supplement-Figure_S1.jpg]

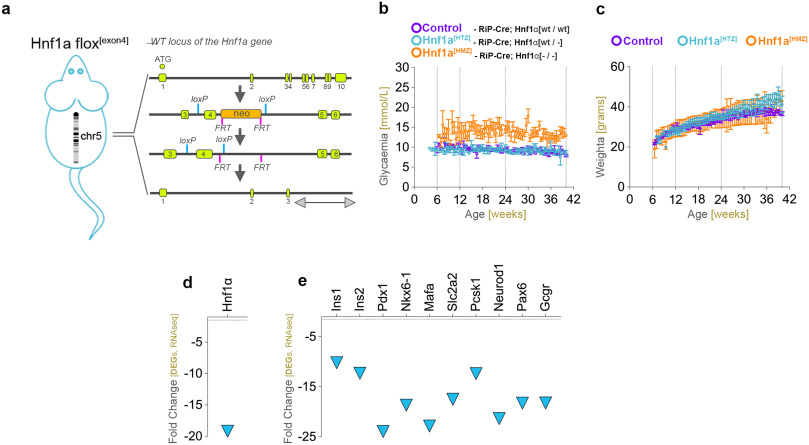

Supplement: Figure S2 [file NIHMS2123777-supplement-Figure_S2.jpg]

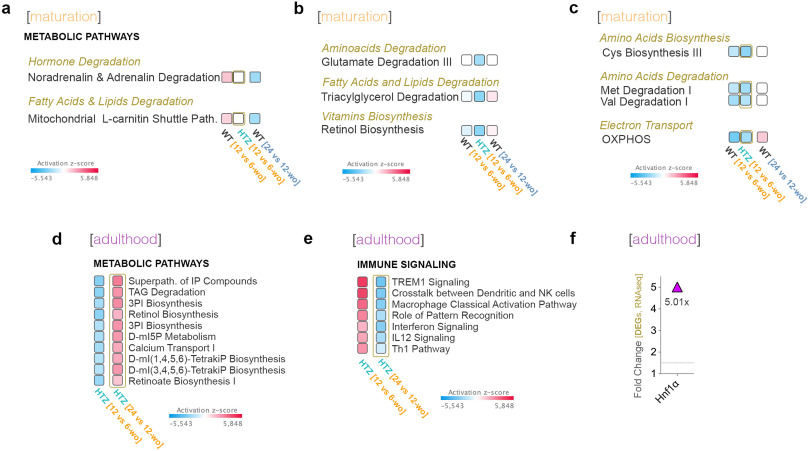

Supplement: Figure S3 [file NIHMS2123777-supplement-Figure_S3.jpg]

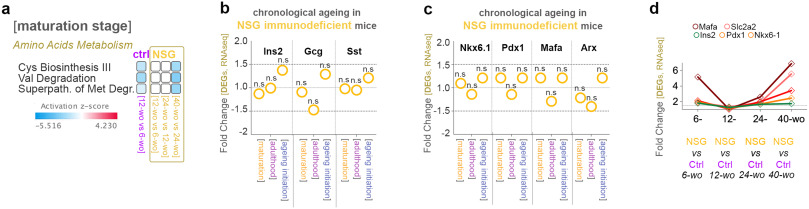

Supplement: Figure S4 [file NIHMS2123777-supplement-Figure_S4.jpg]
